# Intrinsic i‐Wave Altermagnetism in 2D Graphene Superlattices

**DOI:** 10.1002/advs.78167

**Published:** 2026-10-20

**Authors:** Cuiju Yu, Jose L. Lado

**Affiliations:** ^1^ Department of Applied Physics Aalto University Espoo Finland

**Keywords:** graphene superlattices, higher‐order altermagnetism, metal‐free spintronics

## Abstract

Altermagnets feature unconventional magnetism due to their momentum‐dependent spin splitting purely driven by magnetic order, for which a variety of transition‐metal‐based d‐wave altermagnets have been proposed. However, carbon‐based altermagnets remain largely unexplored, even though magnetism of graphene nanostructures has been widely demonstrated. Here, through a comprehensive symmetry analysis, we establish a symmetry‐guided design principle for realizing higher‐order i‐wave altermagnetism in graphene antidot superlattices and demonstrate its emergence in both monolayer and bilayer graphene, particularly in the Bernal‐stacked bilayer. By combining atomistic tight binding models and first principles methods, we show the appearance of an interaction‐induced i‐wave altermagnetic splitting, originating from the intrinsic magnetic instability of 2D graphene antidot superlattices. Our work establishes a strategy to engineer i‐wave altermagnetism in a graphene platform, extending the materials landscape beyond conventional d‐wave altermagnets and putting forward a carbon‐based platform for higher‐order altermagnetic spintronics.

## Graphical abstract

Altermagnetism has so far been mostly proposed in transition metals compounds. Here we show that monolayer and bilayer graphene antidot superlattices realize a carbon‐based i‐wave altermagnet. This is demonstrated with a combination of symmetry analysis, first principles methods and effective models, featuring a six‐fold spin splitting texture in momentum space.

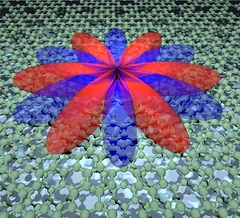

## 1 Introduction

Altermagnets (AMs) provide flexible platforms to explore unconventional magnetic order, simultaneously sharing properties of ferromagnets and antiferromagnets [1, 2, 3, 4, 5]. AMs combine vanishing net magnetization and negligible stray field [6], as in antiferromagnets with fully compensated magnetic moment, with spin‐split band structures, as in ferromagnets [7]. AMs hold great promise for realizing nontrivial topological states [8, 9, 10, 11], and energy‐efficient, highly integrated spintronic devices [12, 13], attracting considerable attention in condensed matter physics and spintronics. AMs can be classified according to the symmetry of the spin‐splitting in momentum space, leading to d‐wave, g‐wave, and i‐wave types [14], similar to the classification of superconducting order parameters. Among these, d‐wave altermagnets, typically realized in tetragonal rutile structures, such as RuO_2_ [12, 15], have been extensively explored so far [16]. Such systems usually exhibit pronounced anisotropy of the effective masses along the $k_{x}$ and $k_{y}$ axes in momentum space, which translates into direction‐dependent spin currents [17, 18]. In such systems, ideal altermagnetic phases can be engineered through diverse structural‐engineering strategies, for instance interlayer sliding [19], Janus structures [18] and twisting [20]. Interestingly, dual‐switchable multiferroic altermagnets  [21, 22, 23, 24, 25] have emerged as a rapidly developing route to electrically and structurally control spin splitting. Besides the intrinsic altermagnets, extrinsic altermagnets can be realized by applying external stimuli, such as electric fields [26] and mechanical strain [27], further enriching the design routes and enhancing the tunability of altermagnetic states, for example in RuO_2_‐based spin‐orbit torque devices [28]. Regardless of specific systems, altermagnetism typically arises when opposite spin sublattices are related by spin space group symmetries, such as rotation, mirror, screw, rather than by translation or inversion [29].

Existing proposals for achieving altermagnetism are largely confined to systems containing transition metals [16, 18, 30, 31, 32, 33], and realizations on metal‐free platforms are almost unexplored. Graphene‐based systems have risen as a flexible playground to engineer magnetism [34]. In particular, ferromagnetic states naturally arise in zigzag graphene nanoribbons [35, 36, 37, 38], giving rise to large magnetically‐induced exchange splittings [39]. Single hydrogenation of graphene gives rise to local S=1/2 magnetic moments [40], providing a highly tunable platform to engineer controllable magnetic superlattices by controlling hydrogenation with scanning tunneling microscopy. Furthermore, nanographene systems [41, 42, 43, 44, 45] provide a platform to create effectively zero and one‐dimensional magnetic systems, enabling the realization of paradigmatic models of quantum magnetism [46, 47], including topologically ordered states [48, 49]. Furthermore, graphene multilayer systems have been shown to lead to magnetically ordered states stemming from valley polarization, both in the case of twisted multilayers [50, 51] and rhombohedral multilayers [52, 53, 54]. Despite the diverse realizations of graphene magnetism, metal‐free altermagnetism has only recently been proposed in nanographene frameworks  [55] and H‐engineered monolayer graphene  [56]. Building on these advances, our design realizes a higher‐order i‐wave altermagnetism and further generalizes this symmetry class to bilayer graphene, particularly the previously unexplored Bernal‐stacked configuration. A higher‐order i‐wave state is also not detected in the same way as a d‐wave one, since the linear responses used for d‐wave altermagnets cannot detect an l=6 form factor, and momentum‐resolved probes, such as the chiral magnon should be used instead [57, 58, 59].

Here we demonstrate the emergence of i‐wave altermagnetism in graphene antidot superlattices, where magnetism stems purely from carbon. We propose a design rule for a 2D metal‐free hexagonal lattice, achieving unconventional i‐wave altermagnetism both in monolayer and bilayer graphene superlattices. We show that the crucial ingredient is to design an antidot superstructure with three‐fold rotational symmetry in which every mirror of the lattice exchanges the two spin sublattices, which accommodates i‐wave altermagnetism. We demonstrate this strategy by combining atomistic tight binding models with first‐principles density functional theory methods, showing the robustness of the i‐wave altermagnetic state, in particular against lattice relaxations. Our manuscript is organized as follows. In Section 1, we provide an overview of recent advances in altermagnetism and nanographene systems. In Section 2, we detail general design principles in graphene antidot superlattice, followed by a mean‐field Hubbard model validation and material realization via first principles simulations to demonstrate i‐wave altermagnetism in graphene antidot superlattices.

## 2 Graphene Altermagnetic Antidot Superlattice

Monolayer graphene has a non‐magnetic ground state, although its electronic interactions put it at the verge of an electronic instability [62]. Graphene multilayers, including aligned bilayer graphene, develop such an electronic instability [63, 64, 65, 66]. The essence of our strategy is to leverage such electronic instability in a modified graphene geometry, either in monolayer or bilayer, whose lattice symmetries give rise to an unconventional altermagnetic i‐wave order (Figure 1a). This is realized by a set of $C_{3}$ antidots arranged in a pattern whose mirror planes all exchange the two magnetic sublattices, leading to an antidot graphene superlattice [67, 68, 69, 70, 71], as shown in Figure 1b. From the perspective of the spin symmetry group, the present model belongs at least to the $C_{3z}$ symmetry together with a spin‐exchanging symmetry, which accommodates the presence of i‐wave altermagnetism. The overall planar six‐fold symmetry elements of the antidot lattice, realized through rotational or rotoinversion operations, produce an effective honeycomb network, essential for i‐wave spin‐momentum locking, as detailed below. It is worth noting that there are no unpaired electrons in the structure, as each dot arises from the removal of two sites in the original honeycomb lattice.

**FIGURE 1:**
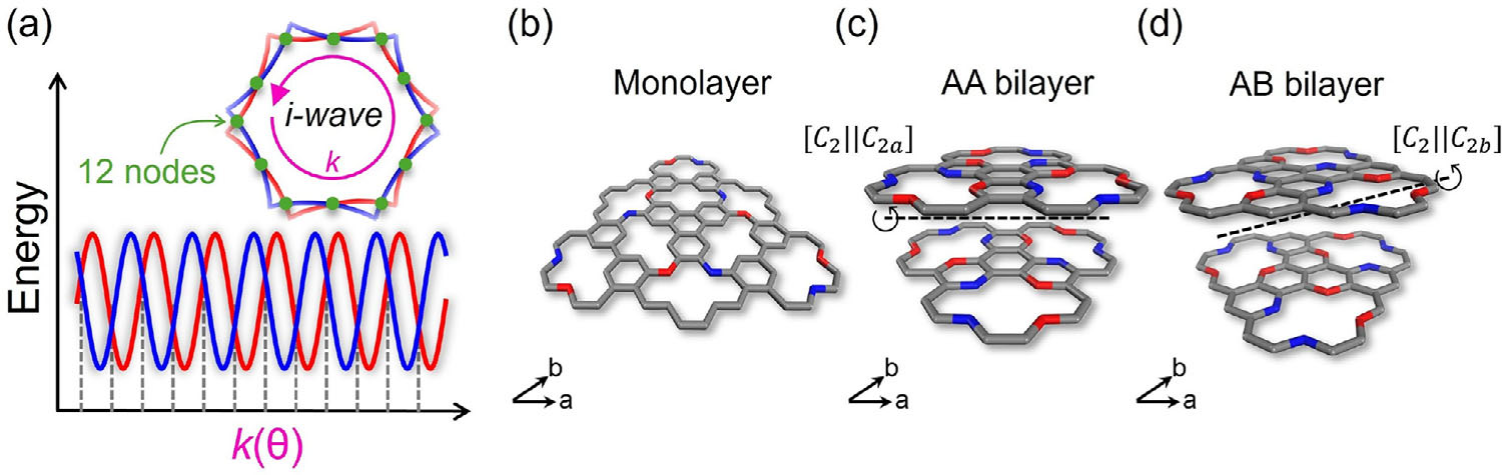
(a) Schematic representation of planar i‐wave spin‐momentum locking character protected by spin‐point group symmetries across reciprocal space. Also shown is the spin‐split band structure of the proposed i‐wave altermagnet. Dashed gray lines drawn through the crossing points mark 12 nodes over the angular range from 0 to 2π, consistent with the i‐wave altermagnetism. (b–d) Schematic of the divacancy‐induced i‐wave altermagnetism in monolayer and bilayer regimes. Panel (b) shows a divacancy‐engineered monolayer with spin point symmetry 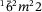 in 2D k‐space, in the notation of Šmejkal [60, 61]. Panels (c,d) depict symmetrically arranged antidots in the AA‐stacked (c) and AB‐stacked (d) bilayers, giving rise to planar spin point group symmetries 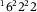 (or 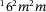) and 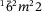, respectively. Here, red and blue short bars indicate local up and down magnetization.

From the experimental point of view, such systems can be constructed in two different ways. A first option is a bottom‐up synthesis of 2D nonplanar triple‐porous nanographene with three‐fold symmetry on metal substrates, which has been demonstrated with on‐surface reactions, such as cyclodehydrogenation [72, 73, 74, 75], yielding a structural framework closely resembling that of our proposed modified graphene. A second strategy relies on effectively removing electronic $p_{z}$ orbitals in selected carbon sites from hydrogen adatoms, as demonstrated using atomic manipulation with scanning tunneling microscope (STM) [40, 56]. Specifically, when a single hydrogen adatom is added on top of a carbon site, the π‐electron of carbon forms a covalent bond with H, leading to an effective removal of the $p_{z}$ from the underlying low energy model [76, 77, 78]. This fabrication strategy has the advantage that no chemical synthesis is needed, with the limitation that creating a bilayer structure can be more challenging.

Lieb's theorem [79] implies that total spin magnetization is strictly zero when the numbers of A‐ and B‐sublattice sites are equal for the Hubbard model in a bipartite lattice. The intrinsic correlated antiferromagnetic (AFM) ground state in the antidot superlattice thus satisfies this constraint for achieving altermagnetism [80]. Analysis of 20 2D centrosymmetric and non‐centrosymmetric spin point groups (PG) identifies four candidates for planar i‐wave altermagnetism: 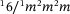, 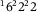, 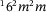 and 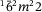 [60, 61]. While a $C_{6}$ symmetry constraint can be readily realized in the monolayer limit through artificial divacancies, spin is intrinsically locked to the real‐space orientation of antidots because of its AFM nature, thus yielding the 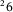 symmetry, instead of our desired 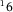. In this way, in the monolayer limit, the first three candidates are excluded from realizing the i‐wave character. Interestingly, the remaining case of 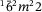 makes i‐wave altermagnetism plausible as it belongs to a reduced symmetry relative to the other three cases while remaining compatible with the AFM nature in antidot superlattices. As illustrated in Figure 1b, a notable finding is that 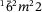 can be realized by deliberate engineering of $P\bar{6}$‐symmetric antidots in a monolayer superlattice. This requires antidots to exhibit six‐fold rotoinversion symmetry corresponding to PG $C_{3h}$ with identity operation in spin space (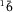), while preserving a mirror plane and an in‐plane two‐fold axis, each accompanied by a two‐fold rotation in spin space (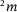 and 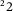), so that the two opposite‐spin sublattices are related by these operations rather than by translation or inversion. It should be noted that the highest symmetry retained by the spin arrangement is only 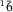, rather than 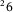.

Going beyond the monolayer limit, we generalize our strategy to bilayer graphene (BG). BG hosts interlayer AFM correlations due to Lieb's theorem, which stabilizes an antiparallel spin arrangement [81]. Taking advantage of the layer degree of freedom, we focus on two representative stacking forms, AA and AB (Bernal), to figure out how to realize intrinsic i‐wave altermagnetism. Since two monolayers are perfectly aligned in the AA‐stacked configuration, a planar i‐wave pattern can be obtained by tailoring six pairs of antidots along hexagonal edges of graphene. In other words, the opposite spin lattices are connected by a $C_{2a}$ rotation, which maps the $C_{3}$‐symmetric antidot pattern in the upper (lower) layer onto the lower (upper) layer (Figure 1c), generating six evenly distributed petals with 12 nodal points (Figure 1a). Meanwhile, the intra‐ and inter‐layer AFM correlations maintain antiparallel in‐plane magnetic orders in the $C_{6}$ symmetric antidot lattice, thereby sustaining the planar spin‐momentum locking of the spin PG 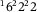 (Figure 1c) and achieving the i‐wave altermagnetism [60, 61]. For AB stacking, it can be viewed as in‐plane sliding of AA stacking. However, the resulting shift of rotation center completely breaks the whole symmetry when the same strategy as for AA stacking is applied. From the symmetry analysis, the first three spin PG candidates are ruled out by lower $D_{3d}$ symmetry of pristine AB stacking, rather than $D_{6h}$ in AA. This leaves 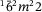 as allowed spin PG to realize i‐wave altermagnetism. As shown in Figure 1d, the antidot pairs are arranged to respect a six‐fold rotoinversion axis in each layer, thereby satisfying 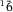 symmetry, while 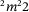 can be solely generated by in‐plane $C_{2b}$ symmetry, thus the overall planar 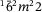 spin PG symmetry is successfully encoded into Bernal‐stacked graphene superlattice. Beyond the spin point‐group analysis, we also perform a rigorous and complete spin space group (SSG) analysis (Table S1; see Supporting Information for details).

### 2.1 Altermagnetism From Self‐Consistent Mean‐Field Calculations

To demonstrate the emergence of altermagnetism in those graphene superlattices, we perform self‐consistent calculations of an atomistic mean‐field model. We describe electronic structures of the graphene antidot superlattice with a Hamiltonian of the form [82]

(1) $$H=H_{0}+H_{U},$$

where $H_{0}$ is the non‐interacting hopping term $H_{0}=\sum_{ij,\sigma }t_{ij}\;c_{i\sigma }^{†}c_{j\sigma }$ and $H_{U}$ is the local electrostatic interaction $H_{U}=U\sum_{i}c_{i↑}^{†}c_{i↑}c_{i↓}^{†}c_{i↓}$, where $t_{ij}$ are the hoppings and U is the on‐site Hubbard interaction. $c_{i\sigma }^{†}$ and $c_{i\sigma }$ correspond to the creation and annihilation operators of spin σ on site i, respectively. The parameter $t_{ij}$ represents hopping amplitude between different sites, which include the intralayer hopping t and the interlayer coupling $t_{⊥}$. We set the intralayer nearest‐neighbor hopping to t = 2.7 eV and the interlayer nearest‐neighbor hopping to $t_{⊥}$ = 0.81 eV. We take a local Hubbard interaction of U=2t=5.4 eV [62]. It is worth noting that for a pristine monolayer, such parameters give rise to a non‐magnetic ground state as expected in pristine graphene. In contrast, an antidot superlattice will have an effective lower Fermi velocity, giving rise to a correlated state at the realistic interaction. The i‐wave splitting persists for U above critical interaction $U_{c}/t\approx 0.35$–0.65 and is insensitive to second‐nearest‐neighbor hopping $t_{2}$ (Figures S1–S3). The interacting term $H_{U}$ is solved at the Hartree‐Fock mean‐field level, using a decoupling $n_{i↑}n_{i↓}\approx \left\langle n_{i↑}\right\rangle n_{i↓}+\left\langle n_{i↓}\right\rangle n_{i↑}-\left\langle n_{i↑}\right\rangle \left\langle n_{i↓}\right\rangle$, yielding the self‐consistent mean‐field Hamiltonian $H_{\mathrm{MF}}$ parameterized by the local spin‐resolved densities [83], which takes the form

(2) $$H_{\mathrm{MF}}=\sum_{ij,\sigma }t_{ij}\;c_{i\sigma }^{†}c_{j\sigma }+\sum_{i,s,s^{\prime }}{\vec{J}}_{i}\cdot {\vec{\sigma }}_{s,s^{\prime }}c_{is}^{†}c_{is^{\prime }}$$

where ${\vec{J}}_{i}$ is the interaction‐induced local exchange field, associated with the emergence of a local magnetic order ${\vec{M}}_{n}=\sum_{s,s^{\prime }}{\vec{\sigma }}_{s,s^{\prime }}\left\langle c_{n,s}^{†}c_{n,s^{\prime }}\right\rangle$. Due to the bipartite nature of the system, the mean‐field ground state develops AFM order as given by Lieb's theorem [79], and the self‐consistent magnetization is taken along the *z*‐axis ${\vec{M}}_{n}=\left(0,0,M_{n}^{z}\right)$.

The appearance of altermagnetism can be directly quantified by the average altermagnetic order defined as $\Delta =\int d^{2}k\sum_{\alpha }\sqrt{{\left(\varepsilon_{\alpha ,↑}-\varepsilon_{\alpha ,↓}\right)}^{2}}$ where $\varepsilon_{\alpha ,s}$ is the eigenvalue α for the spin channel s. The previous definition can be extended to the frequency range to quantify which energies contribute the strongest to the altermagnetic order, by means of the altermagnetic spectral density defined as

(3) $$\Xi \left(\omega \right)=\int d^{2}k\sum_{\alpha }\sqrt{{\left(\varepsilon_{\alpha ,↑}-\varepsilon_{\alpha ,↓}\right)}^{2}}\delta \left(\omega -\frac{\varepsilon_{\alpha ,↑}+\varepsilon_{\alpha ,↓}}{2}\right)$$

where δ is the Dirac delta function. By definition we have Δ=∫Ξ(ω)dω.

We first present the results for a monolayer graphene antidot superlattice (Figure 2a). Self‐consistent mean‐field (SCMF) calculations give rise to a magnetic ground state, for which the spin density distribution confirms that the ground state is AFM induced by the electronic instability (Figure 2b). Subsequently, we analyze spin‐resolved electronic bands by computing the $\left\langle S_{z}\right\rangle$ expectation value of each eigenstate. We take a momentum space loop around the Γ point in the Brillouin zone as $k\left(\theta \right)=k_{0}\left(\cos\theta ,\sin\theta ,0\right)$, where $k_{0}=0.3\left|b_{1}\right|$ with $|b_{1}|$ the reciprocal lattice vector. A conventional Γ–K–M–Γ path would not show a splitting, because the twelve nodal directions of an l=6 form factor are spaced by $30^{\circ }$ and the Γ–M and Γ–K directions of a hexagonal Brillouin zone are separated by exactly that angle, so both radial segments of that path are nodal. A loop of fixed |k| instead sweeps the angle and exposes the six‐fold sign alternation directly. As shown in Figure 2c, the bands clearly show spin splitting in the reciprocal space, oscillating along the angular k‐path coordinate θ. Within the angular range θ∈[0,2π], twelve nodes (six pairs) of alternating spin splittings are observed, consistent with the six‐fold symmetry of i‐wave altermagnetic order in Figure 1a. A harmonic decomposition confirms this assignment, with only the sixth‐order component surviving (Figure S4). By quantifying the altermagnetic order, we observe that its density peaks at 1.17t (Figure 2d). To further illustrate the emergence of i‐wave order, we examine the Fermi surface at ω=1.17t, as shown in Figure 2e. The symmetrically distributed, petal‐like spin‐up and spin‐down Fermi surfaces further confirm the presence of twelve nodal points in the Brillouin zone, consistent with split‐ring features (Figure 2c).

**FIGURE 2:**
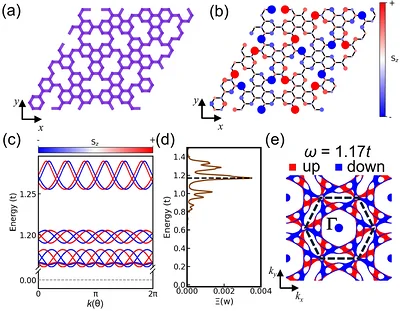
(a) Top view of an antidot $6\sqrt{3}\times 6\sqrt{3}$ monolayer graphene superlattice. (b) Self‐consistent spin density distributions. Here, red and blue solid circles represent spin up and spin down moments, respectively, and their sizes indicate the magnitudes of magnetic moments. (c) Spin‐resolved band structure hosting spin‐splitting character, in a path centered at the Γ point of the Brillouin zone. Positive and negative values on the color bar denote spin‐up and spin‐down $S_{z}$ components, respectively. The intralayer nearest‐neighbor hopping is taken as the energy unit. The gray dashed line indicates the Fermi level. (d) Altermagnetic order density Ξ(ω) versus energy. (e) Spin‐resolved Fermi surface at ω=1.17t corresponding to the dashed line in (d).

We now address the emergence of altermagnetism in bilayer superlattices. We first consider the antidot superlattice in AA‐stacked configuration depicted in Figure 1c. We perform SCMF calculations of the bilayer model (Figure 3a), for which the spin density distribution of upper and lower layers reveals the emergence of a superlattice modulated antiferromagnetic order (Figure 3b). Subsequently, we analyze the spin‐resolved electronic band structure. As shown in Figure 3c, the bands exhibit a spin‐dependent splitting in the reciprocal space, distributed along the angular coordinate θ. For θ∈[0,2π], six pairs of crossing splitting rings are observed, consistent with 12 nodal points in Figure 1a. As shown by the altermagnetic spectral density in Figure 3d, a sizable spin splitting peak emerges at ω=0.9t. The altermagnetic i‐wave character can be directly observed on the Fermi surface (Figure 3e). The spin‐split Fermi surfaces alternate between hexagonal vertices in k‐space and exhibit 12 nodes.

**FIGURE 3:**
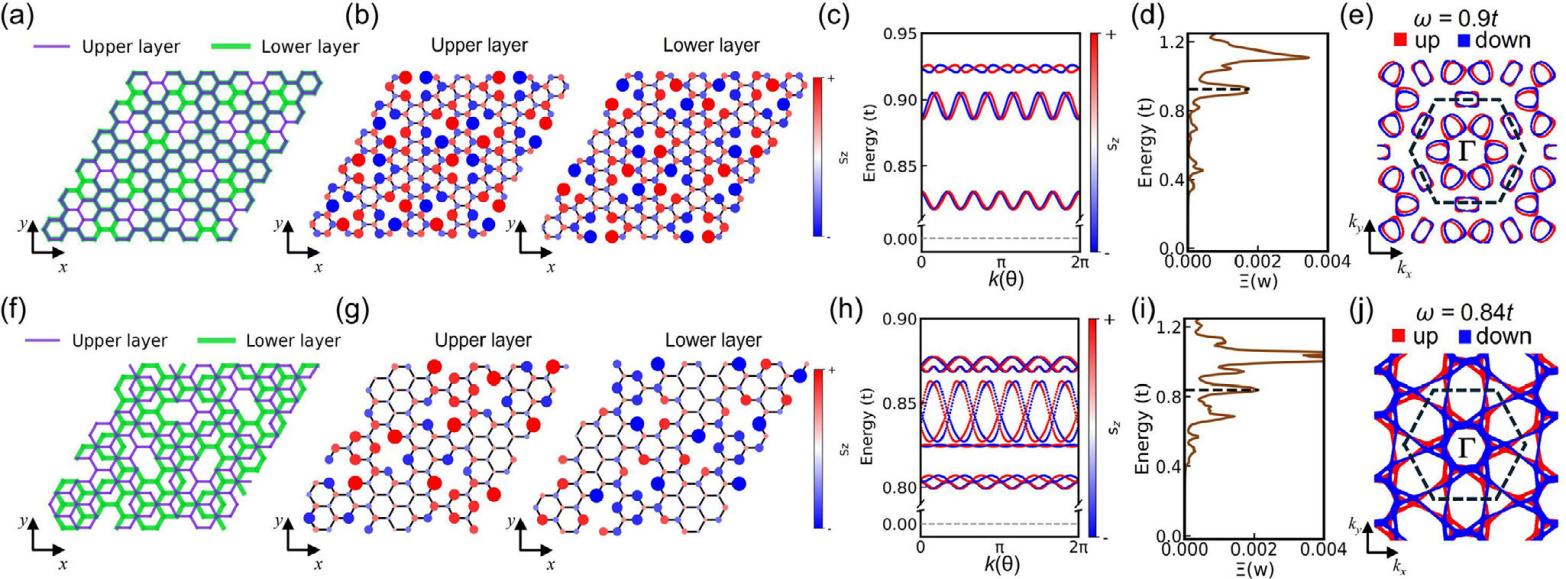
(a,f) Top‐view schematics of antidot structures in $3\sqrt{3}\times 3\sqrt{3}$ AA‐stacked, $5\sqrt{3}\times 5\sqrt{3}$ Bernal‐stacked graphene superlattices, respectively. (b,g) Corresponding self‐consistent spin density distributions in top and bottom layers. Red and blue solid circles denote spin‐up and spin‐down magnetic moments, respectively, with their sizes proportional to the magnitudes of the actual local magnetic moments. (c,h) Spin‐resolved band structures showing spin splitting for AA and AB stackings, respectively, in a path centered at the Γ point of the first Brillouin zone. Here, the gray dashed lines indicate the Fermi level. (d,i) Altermagnetic order density Ξ(ω) as a function of energy for AA and AB stackings, respectively. Here the black dashed line marks the local maximum. (e,j) Spin‐resolved Fermi surfaces centered at Γ point for AA and AB stackings at ω=0.9t and ω=0.84t, corresponding to the local maximum values in (d) and (i), respectively. It should be noted that, since the energy bands are exactly symmetric about the Fermi level, only the positive‐energy bands are shown.

We now consider the Bernal‐stacked AB bilayer antidot superlattice. The spin space symmetry of the BG antidot superlattice belongs to the 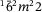, where the top view is shown in Figure 3f. The resulting mean‐field spin density distributions confirm the emergence of superlattice‐modulated antiferromagnetic correlations between sublattices in the ground state (Figure 3g). From the band structure in a loop around the Γ point, one clearly sees alternating spin‐splitting loops, with 12 nodal points along the k‐path in the range θ∈[0,2π] in Figure 3h. The frequency‐resolved altermagnetic order density shown in Figure 3i shows a sizable altermagnetic ordering at the energy ω=0.84t, as observed in Figure 3h. The spin Fermi surface resembles two sets of distorted six‐pointed stars, confirming the alternating‐loop character of the electronic band and exhibiting 6‐fold altermagnetic splitting in first Brillouin zone (Figure 3j). As a finite Néel temperature is not well defined for an ideal 2D antiferromagnet with negligible spin‐orbit coupling (SOC), we instead extract the spin stiffness from the RPA magnon velocity (Figure S5; see Supporting Information for details), obtaining $\rho_{s}\approx 160$–180meV for the monolayer and bilayers, six to seven times the room‐temperature thermal energy, which indicates robust short‐range antiferromagnetic correlations. The weak spin‐orbit anisotropy of graphene cuts off the thermal magnon divergence and turns this stiffness into an estimated ordering temperature of about 245 K for the monolayer and 225 K for bilayers, well below the local‐moment formation temperature (Figure S6; see Supporting Information for details).

### 2.2 Altermagnetism From First Principles

Density functional theory (DFT) calculations were performed using the Vienna ab initio Simulation Package (VASP) [84, 85]. The interaction between electrons and ions was described using the projector‐augmented‐wave (PAW) method [86, 87], while exchange‐correlation effects were accounted for within the generalized gradient approximation (GGA) [88]. For structural optimization, the Perdew–Burke–Ernzerhof (PBE) functional is employed for computational efficiency. The energy cutoff was set to 600 eV for the plane wave basis set, with a dense 6×6×1 k‐point mesh. The supercell structure was fully relaxed until the residual forces were below 0.001 eV/Å, and the change of total energy was less than 10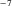 eV. The van der Waals interaction was accounted for using the DFT‐D3 method for monolayer and bilayer graphene [89]. A vacuum layer of 17.0 Å was introduced along the c‐axis to prevent periodic interactions. Within both self‐consistent and non‐self‐consistent calculations, we employed the meta‐GGA SCAN functional [90], which is able to obtain a band gap closer to experiment. In pristine BG, the calculated band gap using SCAN functional is 8.3 meV, in reasonable agreement with experimentally reported values of about 8 or 2.5 meV [65, 91]. Thermal and dynamical stability was evaluated by ab initio molecular dynamics (AIMD) and phonon calculations (Figures S7 and S8).

As a proof of concept of i‐wave altermagnetism for the aforementioned antidot‐engineered superlattices, we construct three representative material realizations, namely hydrogen‐passivated vacancy superlattices in monolayer graphene, and in AA‐ and Bernal‐stacked BG. First, we investigate structural, magnetic and electronic properties of the monolayer graphene antidot superlattice. Starting from 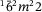 symmetry in the unit cell with antidots, we find that the fully relaxed structure deviates from planarity and exhibits an out‐of‐plane distortion of about 2.44 Å, as shown in Figure 4a. To further evaluate the magnetic ground state, total energies of nonmagnetic and AFM supercell configurations are analyzed. The AFM state lies 2.96 eV below the nonmagnetic state, establishing it as the ground state. The electronic band structure exhibits momentum‐dependent spin splitting with 12 nodal points over the angular range θ∈[0,2π] (Figure 4c,d). The split states are dominated by C $p_{z}$ orbitals (Figure S9), with the spin density delocalized across the lattice (Figure 4b). Despite hydrogen‐induced spatial variations in the hopping and exchange fields, the out‐of‐plane buckling constitutes a symmetry‐allowed structural distortion that preserves the characteristic symmetric 12‐node i‐wave pattern (Figure 1a). The spin splitting of the buckled structure is comparable to that of the planar geometry (Figure S10). The resulting spin‐momentum locking is a defining hallmark of i‐wave altermagnetism. It is worth noting that the antidot‐engineered supercell closely resembles porous graphene nanoribbons, where hydrogen‐passivated vacancies are commonly adopted experimentally to stabilize the bare carbon edges. The same three‐fold‐symmetric antidot arrangement in nonplanar triple‐porous graphene nanostructures has been realized and characterized by STM and atomic force microscopy, which strongly supports the feasibility of our proposal [73, 75].

**FIGURE 4:**
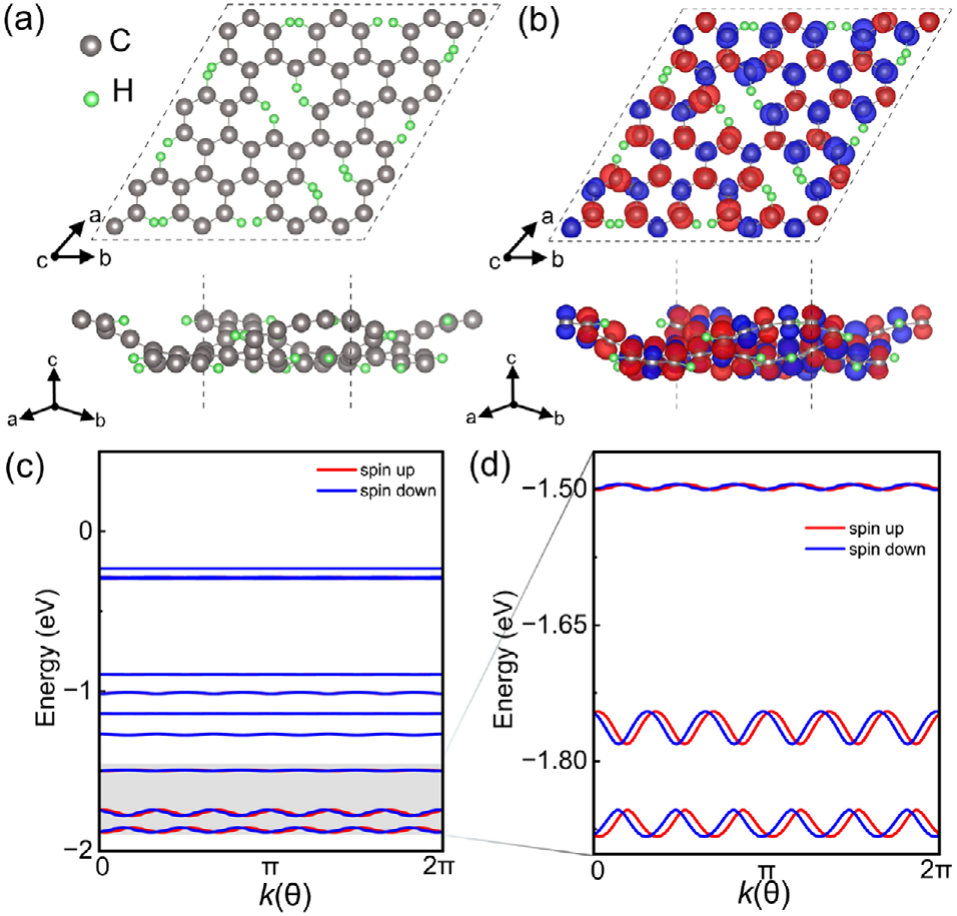
(a) Schematic top and side views of divacancy‐hydrogenated monolayer graphene of 6×6 supercell. (b) Top and side views of spin density distribution in the ground state, where red and blue denote spin‐up and spin‐down densities. (c) Spin‐resolved band structure along a circular path over the angular range from 0 to 2π. (d) Enlarged view of the spin‐split bands in the highlighted region of (c).

We then turn to bilayer graphene antidot superlattice with AA‐ and Bernal‐stacked configurations. For the AA antidot superlattice in the minimal graphene supercell (Figure 5a), the fully optimized structure develops a buckled geometry along the *z* axis, indicative of structural distortion driven by hydrogen‐induced rehybridization from $sp^{2}$ to $sp^{3}$. To verify the magnetic ground state, three configurations were examined, nonmagnetic, interlayer ferromagnetic with intralayer AFM, and interlayer AFM with intralayer AFM. Their relative total energies are consistent with an AFM ground state (Figure 5b), as expected from Lieb's theorem. In addition to the magnetic ground state, the electronic band structure exhibits a large band gap of 1.96 eV, confirming the insulating nature of the system, consistent with the gapped ground state of the model above. The spin‐resolved band structure reveals alternating splitting from 0 to 2π in reciprocal space (Figure 5c,d). Notably, within the energy window from −1.20 to −1.14 eV, the intersecting spin‐split loops form a six‐fold pattern with 12 nodal points along a uniformly sampled circular path in the Brillouin zone, which serves as a hallmark of i‐wave altermagnetism. This result is consistent with the Hubbard‐model prediction and further confirms that the symmetry‐allowed 1.63 Å buckling within each individual graphene layer preserves the essential i‐wave structure. Despite this structural buckling reconstruction, the i‐wave altermagnetic order follows the results of the minimal atomistic model described previously. The buckling is expected to decrease in heterostructures encapsulated in hBN, as observed in other graphene devices [92].

**FIGURE 5:**
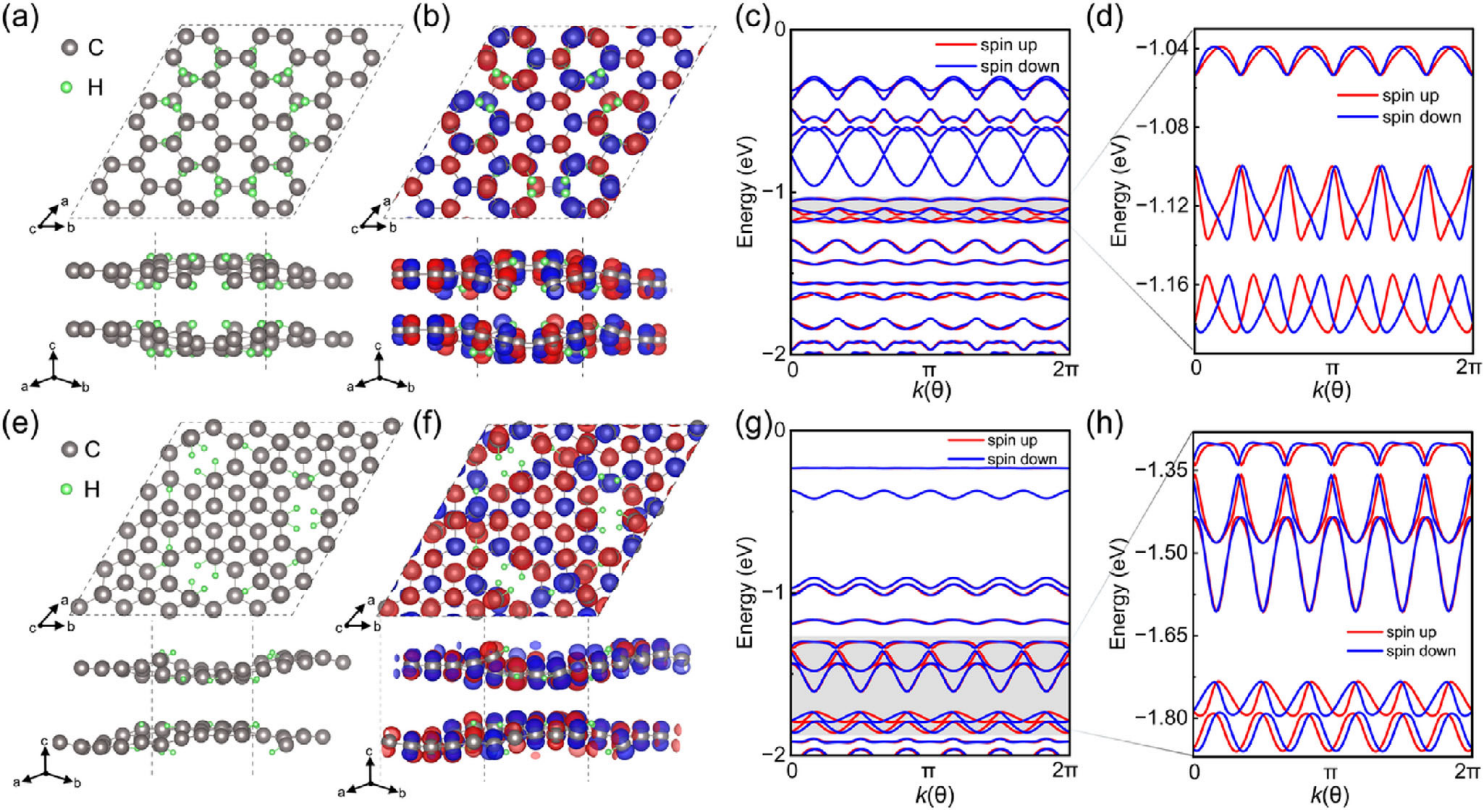
(a,e) Schematic top and side views of graphene superlattices, stemming from $3\sqrt{3}\times 3\sqrt{3}$ AA‐stacked and 5×5 Bernal‐stacked graphene supercells, respectively. (b,f) Top and side views of spin density distribution in ground states of AA and AB stackings, respectively, where red and blue shadows separately denote spin‐up and spin‐down densities. (c,g) Spin‐resolved band structures within the angular range from 0 to 2π of a path around the Γ point for AA and AB stackings, respectively. (d,h) Enlarged views of spin‐split bands in gray highlighted regions of (c) and (g) for AA and AB stackings, respectively.

We now address the Bernal‐stacked antidot superlattice in the minimal graphene supercell as shown in Figure 5e. From a structural perspective, the Bernal‐stacked antidot superlattice is energetically favored over the AA‐stacked one by about 20 meV per superlattice unit cell. The AA‐stacked configuration is nevertheless experimentally accessible, both as an extended phase in epitaxial bilayer graphene [93] and as locally AA‐stacked regions of marginally twisted bilayer graphene [94] (see Supporting Information for details). The structure also undergoes out‐of‐plane buckling to relieve local strain, similar to the AA‐superlattice case. From the total energy analysis and spin density distribution, the AFM configuration is energetically preferred to both the nonmagnetic and interlayer ferromagnetic states (Figure 5f). We then move on to the electronic properties of divacancy‐hydrogenated Bernal‐stacked bilayer. As shown in Figure 5g,h, within the energy window from −1.87 to −1.35 eV, one clearly sees six spin‐split loops and twelve nodal points along the k‐path, characteristic of i‐wave altermagnetism, robust to relaxation in all three systems (Figure S11), with splittings comparable to those obtained in DFT (Table S2).

In the shaded regions for AB stacking as shown in Figure 5g, the lower two sets of alternating band spin splitting have complementary behaviors, showing a trend similar to that in the monolayer and AA‐stacked cases. In addition, we examine the spin‐split bands closer to the Fermi level (Figure S12). The finite spin‐split states emerge within 0.6 eV below the Fermi level, suggesting the i‐wave character. Moreover, fully relativistic calculations show that SOC induces only negligible magnetic anisotropy and band‐structure modification, leaving the characteristic i‐wave altermagnetic spin splitting essentially unchanged (Figure S13; see Supporting Information for details). This momentum‐dependent spin splitting makes the spin‐resolved spectral weight strongly anisotropic, with the two spin channels inequivalent along selected momentum directions, e.g., π/12, where the spin splitting reaches its maximum in band structures in Figures 4, 5, and equivalent along the nodal directions. A charge‐transport measurement is nevertheless not the appropriate probe of the net i‐wave order, due to the insulating nature of the ground state. The signature to target instead is the magnon spectrum, as it can be observed in a magnetic insulator. In a compensated collinear altermagnet, the two chiral magnon branches acquire a momentum‐dependent splitting that inherits the angular form factor of the electronic spin splitting. Consequently, the i‐wave electronic structure is directly reflected in the magnon spectrum as a six‐fold‐periodic, sign‐alternating splitting with twelve nodal directions in momentum space (Figure S14). This is accessible to magnon spin‐transport measurements through the magnon spin Nernst and Seebeck effects in a collinear honeycomb geometry closely related to ours [59]. The electronic spin splitting that sets its form factor is directly accessible to spin‐resolved angle‐resolved photoemission spectroscopy (spin‐ARPES) as demonstrated for altermagnetic MnTe [7] and CrSb [95], a technique that reaches states several eV below the Fermi level [96], with micrometer‐focused photoemission now resolving the bands of exfoliated graphene devices [97]. Being nonrelativistic, the splitting requires no spin‐orbit coupling, which suits a carbon platform (see Supporting Information for details).

## 3 Conclusion

In summary, we showed the emergence of momentum‐dependent spin‐splitting associated with i‐wave altermagnetism in graphene antidot superlattices, most notably in Bernal‐stacked bilayer graphene. Using mean‐field Hubbard atomistic models and first principles methods based on density functional theory, we showed that graphene superlattices enable the realization of i‐wave altermagnetism in a pure carbon‐based system. Our mechanism for altermagnetism relies solely on the intrinsic electronic interactions of graphene systems, combined with an engineered arrangement of missing carbon sites realizing an antidot superlattice. Altermagnetism in both monolayer and bilayer systems arises from a spatially modulated antiferromagnetic order triggered by the antidot superlattice driven by electronic interactions, and we demonstrated that the i‐wave order is robust against the presence of atomic relaxations in systems. Our results establish a strategy for engineering metal‐free two‐dimensional altermagnets in nanoengineered graphene systems, extending the material scope of altermagnetism beyond transition‐metal compounds.

## Conflicts of Interest

The authors declare no conflicts of interest.

## Supporting information


**Supporting File**: advs78167‐sup‐0001‐SuppMat.pdf.

## Data Availability

The data and computational results that support the findings of this study are available within the article and its Supporting Information. The relaxed structure files are deposited in Zenodo (DOI 10.5281/zenodo.22658994). Additional data, including relevant computational input files, are available from the corresponding authors upon reasonable request.
