# Supplementary material for: Intrinsic i‐Wave Altermagnetism in 2D Graphene Superlattices
Source: Adv Sci (Weinh). 2026 Oct 20:e78167. Online ahead of print. doi: 10.1002/advs.78167 (PMC13683906; doi:10.1002/advs.78167)
Supplement: Supplementary file 1 [file ADVS-9999-e78167-s001.pdf]

# Supporting Information

## Intrinsic $i$-wave altermagnetism in 2D graphene superlattices

Cuiju Yu*, Jose L. Lado*

Department of Applied Physics, Aalto University, 02150 Espoo, Finland

This Supporting Information provides additional details on the structural, electronic and magnetic properties as well as the benchmarks of the graphene-based $i$-wave altermagnetic systems. We first examine tight-binding parameters, spin space group analysis, Hubbard-$U$ dependence and second-neighbor hopping, angular spin-splitting decomposition, RPA spin response and spin stiffness, and local-moment formation temperature. We then turn to stability verification, orbital-projected DOS, planar, nonplanar and $k$-path-resolved band structures with their nodal degeneracies under relaxation, SOC and magnetic anisotropy, the magnonic detection of the $i$-wave order, supercell selection and symmetry preservation, and the feasibility of AA-stacked bilayer graphene superlattices.

# S1 Tight-binding parameters

The hopping parameters follow standard tight-binding descriptions of graphene. The nearest-neighbor intralayer hopping is taken as $t = 2.7$ eV [1]. The interlayer hopping is modeled using a distance-dependent Slater-Koster parametrization [2], as implemented in Pyqula [3]. For the interlayer spacing $d_z = 3.0$ Å, we use $t_\perp \approx 0.3t \approx 0.81$ eV consistent with an exponential interpolation from $t_\perp(d_z = 3.35\text{ Å}) \approx 0.39$ eV.

For the on-site Hubbard interaction, we use $U = 2t = 5.4$ eV, comparable to the effective value $U_{\text{eff}} \approx 1.6t$ reported for graphene when nonlocal Coulomb interactions are included [4].

# S2 Spin space group analysis

We provide the explicit spin-space-group (SSG) analysis for the monolayer and the AA- and AB-stacked bilayers.

## S2.1 General decomposition

For a compensated collinear magnet, the SSG part can be written as

$$\mathcal{R}_s = [E \parallel H] + [U_\pi \parallel AH] \tag{S1}$$

where $H$ is a halving subgroup of the parent (crystallographic) group $\mathcal{G}$ ($[\mathcal{G}{:}H] = 2$) that preserves each spin sublattice, while $A$ ($\mathcal{G} \setminus H = A\,H$) exchanges the two opposite-spin sublattices, combined with the spin-space $\pi$ rotation $U_\pi$ that flips the spins. The primitive translations $[E \| E \,|\, \mathbf{a}_i]$ are implicitly included. Equation (S1) is a Type-III SSG. It describes an altermagnet, rather than a conventional antiferromagnet, provided that the spin-flip coset contains neither a pure translation nor the spatial inversion, so that the opposite-spin sublattices are linked by a rotation, mirror or screw operation [5, 6].

The spin point groups quoted in the main text are planar spin point groups, not the point-group parts of the three-dimensional SSG decomposition in Eq. (S1). The two are related by restricting each SSG to its action on the $k_x$–$k_y$ plane, and impose the same constraints on the in-plane spin-splitting form factor and hence the same $i$-wave character. In the decomposition below, the superscript-1 operations generate the spin-preserving subgroup $H$ and the superscript-2 operations the spin-exchanging coset $\mathcal{G} \setminus H$.

## S2.2 Monolayer

For the ideal planar monolayer, the spin point group is ${}^{1}\bar{6}\,{}^{2}m{}^{2}2$ (crystal part $D_{3h}$, which contains no spatial inversion), one of the four planar spin point groups that host $i$-wave altermagnetism[7, 8]. The superscripts record which operations are paired with the spin flip. The sixfold rotoinversion $\bar{6}$, and with it the threefold rotation $C_{3z}$ and the horizontal mirror $\sigma_h$, carry the superscript 1 and preserve each spin sublattice, whereas the three vertical mirrors $\sigma_v$ (planes containing the $z$ axis) and the three in-plane twofold axes $C_2'$ carry the superscript 2 and exchange the two opposite-spin sublattices. In the fully relaxed monolayer the structure deviates from planarity (out-of-plane distortion $\sim 2.44$ Å) and the space group is lowered to $P31m$ ($C_{3v}$). Buckling removes $\sigma_h$, and with it $\bar{6}$ and $C_2'$, but the two generators on which the splitting rests survive, namely

$$\left[\,E \,\|\, C_{3z}\,\right] \text{ (spin-preserving)}, \qquad \left[\,U_\pi \,\|\, \sigma_v\,\right] \text{ (spin-exchanging)}, \tag{S2}$$

giving

$$\mathcal{R}_s^{\mathrm{ML}} = \left[\,E \,\|\, P3\,\right] + \left[\,U_\pi \,\|\, \sigma_v P3\,\right]. \tag{S3}$$

Here $C_{3z}$ preserves each spin sublattice, whereas $\sigma_v$ (a vertical mirror plane containing the $z$ axis) exchanges the two opposite-spin sublattices and therefore imposes

$$\Delta E(\sigma_v \mathbf{k}) = -\Delta E(\mathbf{k}).$$

## S2.3 AA- and AB-stacked bilayers

For the AA-stacked bilayer, the space group is $P\bar{3}m1$, which is preserved both before and after buckling. The spin-preserving subgroup is

$$H_{\mathrm{AA}} = P\bar{3},$$

while the remaining operations form the spin-exchanging coset

$$P\bar{3}m1 = P\bar{3} \cup C_{2a}P\bar{3}.$$

The corresponding spin space group is

$$\mathcal{R}_s^{\mathrm{AA}} \;=\; \left[\,E \,\|\, P\bar{3}\,\right] \;+\; \left[\,U_\pi \,\|\, C_{2a}\, P\bar{3}\,\right], \tag{S4}$$

with generators

$$[E \| I C_{3z}], \qquad [U_\pi \,\|\, C_{2a}].$$

Here, the inversion centered on the interlayer midplane exchanges the two layers while preserving the magnetic sublattices, whereas $C_{2a}$ exchanges the opposite-spin sublattices and therefore imposes

$$\Delta E(C_{2a}\mathbf{k}) = -\Delta E(\mathbf{k}).$$

In the AB-stacked bilayer, the Bernal shift reduces the symmetry to the space group $P321$, which is preserved both before and after buckling. The spin-preserving subgroup becomes

$$H_{\mathrm{AB}} = P3,$$

and the spin-exchanging coset is

$$P321 = P3 \cup C_{2b}P3.$$

Accordingly,

$$\mathcal{R}_s^{\mathrm{AB}} = \left[ E \,\|\, P3 \right] + \left[ U_\pi \,\|\, C_{2b}\, P3 \right], \tag{S5}$$

with generators

$$[E \parallel C_{3z}], \qquad [U_\pi \parallel C_{2b}].$$

The spin-exchanging $C_{2b}$ operation similarly gives

$$\Delta E(C_{2b}\mathbf{k}) = -\Delta E(\mathbf{k}).$$

Both bilayers thus share the same spin-symmetry structure, in which $C_{3z}$ preserves the magnetic sublattices, whereas an in-plane twofold rotation, $C_{2a}$ in AA and $C_{2b}$ in AB, exchanges the two opposite-spin sublattices. Combined with $U_\pi$, these operations enforce the sign-changing momentum-dependent spin splitting characteristic of the $i$-wave altermagnetic state. The spin-space-group analysis and the corresponding symmetry generators are summarized in Table S1.

**Table S1:** Summary of the spin-space-group (SSG) symmetry analysis. For each system the first generator preserves the two spin sublattices and the second, combined with the spin flip $U_\pi$, exchanges them.

| **System** | **Space group** | **SSG decomposition** | **SSG Generators** |
|---|---|---|---|
| Monolayer (relaxed) | $P31m$ | $[E\|P3] + [U_\pi\|\,\sigma_v P3]$ | $[E\|C_{3z}]$, $[U_\pi\|\,\sigma_v]$ |
| AA bilayer | $P\bar{3}m1$ | $[E\|P\bar{3}] + [U_\pi\|\,C_{2a}P\bar{3}]$ | $[E\|IC_{3z}]$, $[U_\pi\|\,C_{2a}]$ |
| AB bilayer | $P321$ | $[E\|P3] + [U_\pi\|\,C_{2b}P3]$ | $[E\|C_{3z}]$, $[U_\pi\|\,C_{2b}]$ |

## S2.4 Symmetry operations protecting the $i$-wave state

In the non-SOC limit the Hamiltonian is spin-diagonal, with the splitting $\Delta E(\mathbf{k}) = E_\uparrow(\mathbf{k}) - E_\downarrow(\mathbf{k})$. Three operations fix the angular form of the splitting in all three systems.

- **Spin-exchanging generators** $[U_\pi\|R]$ with $R \in \{\sigma_v, C_{2a}, C_{2b}\}$. They map $(\uparrow, \mathbf{k}) \to (\downarrow, R\mathbf{k})$ and enforce $\Delta E(R\mathbf{k}) = -\Delta E(\mathbf{k})$, so the splitting is odd under a reflection of $\mathbf{k}$ and vanishes on the reflection line.

- **Spin-preserving** $C_{3z} \in \mathcal{H}$. It enforces $\Delta E(C_{3z}\mathbf{k}) = \Delta E(\mathbf{k})$, a period of 120° in the polar angle $\phi$ of $\mathbf{k}$.

- **Spinless time reversal** $S$, the product of time reversal and a spin rotation by $\pi$ about an axis perpendicular to the moments. It is a symmetry of every collinear magnet with real hoppings and negligible spin-orbit coupling, acts within each spin channel as $E_\sigma(\mathbf{k}) = E_\sigma(-\mathbf{k})$, and hence gives $\Delta E(-\mathbf{k}) = \Delta E(\mathbf{k})$, a period of 180° in $\phi$.

The last two together give $\Delta E$ a period of 60°, because the only angular harmonics with both a 120° and a 180° period are $l = 0, 6, 12, \ldots$, and the first removes $l = 0$ because an odd function has no constant part. Every harmonic below $l = 6$ therefore vanishes identically. The lowest-order polynomial compatible with all three constraints is $k^6 \cos 6\phi$, a form factor with an angular period of $\pi/3$ that changes sign every $\pi/6$ and has twelve nodal directions in the Brillouin zone, which is the $i$-wave ($l = 6$) angular dependence [5].

# S3 Hubbard-$U$ dependence and second-nearest-neighbor hopping dependence

In this section $t_1$ denotes the nearest-neighbor intralayer hopping, written as $t$ elsewhere, and $t_2$ the second-nearest-neighbor hopping. We have scanned the Hubbard interaction over $U/t_1 = 0$–$2$ and examined its effect on the local magnetic moment per C site (in $\mu_B$) and on the spin-splitting energy for the three antidot superlattices. Both the maximum local magnetic moment and the spin-splitting energy generally increase with increasing Hubbard $U$. The critical interaction $U_c$ is defined by the onset of a stable self-consistent magnetic solution with a finite local moment. As shown in Fig. S1a,b, the $U$ scan places the onset at $U_c/t_1 \approx 0.41$ (dashed line), marking the transition from the nonmagnetic to the magnetic phase. Similarly, Figs. S2a,b and S3a,b give $U_c/t_1 \approx 0.65$ for the AA bilayer and $U_c/t_1 \approx 0.35$ for the AB bilayer, respectively. In all three systems the moment stays small over a wide interval above the onset and grows rapidly only well above it.

We have also included the second-nearest-neighbor intralayer hopping $t_2$ in the model. For $t_2/t_1 > 0$ the $i$-wave altermagnetic pattern is insensitive to $t_2$, while the positions of the alternating spin-split loops shift, as seen in Fig. S1c-e, Fig. S2c-e and Fig. S3c-e. The spin splitting persists upon inclusion of $t_2$, with its magnitude evolving as $t_2$ increases and the local magnetic moment decreasing slightly. Therefore, we keep only $t_1$ in the minimal model, which captures the essential features of the $i$-wave altermagnetism.

The maximum spin splitting obtained from the tight-binding model and from density functional theory (DFT) is compared in Table S2.

We have also compared the local magnetic moments obtained from the two approaches. The tight-binding model gives 0.255, 0.163 and 0.152 $\mu_B$ per C site for the monolayer, the AA-stacked bilayer and the AB-stacked bilayer, against 0.186, 0.143 and 0.154 $\mu_B$ per C site from the DFT calculations. The model and the DFT calculations use the same supercell only for the AA bilayer, so this comparison is qualitative rather than cell-by-cell. Within that caveat the two approaches yield local moments of the same order of magnitude in all three systems, showing that the model and the DFT description are consistent.

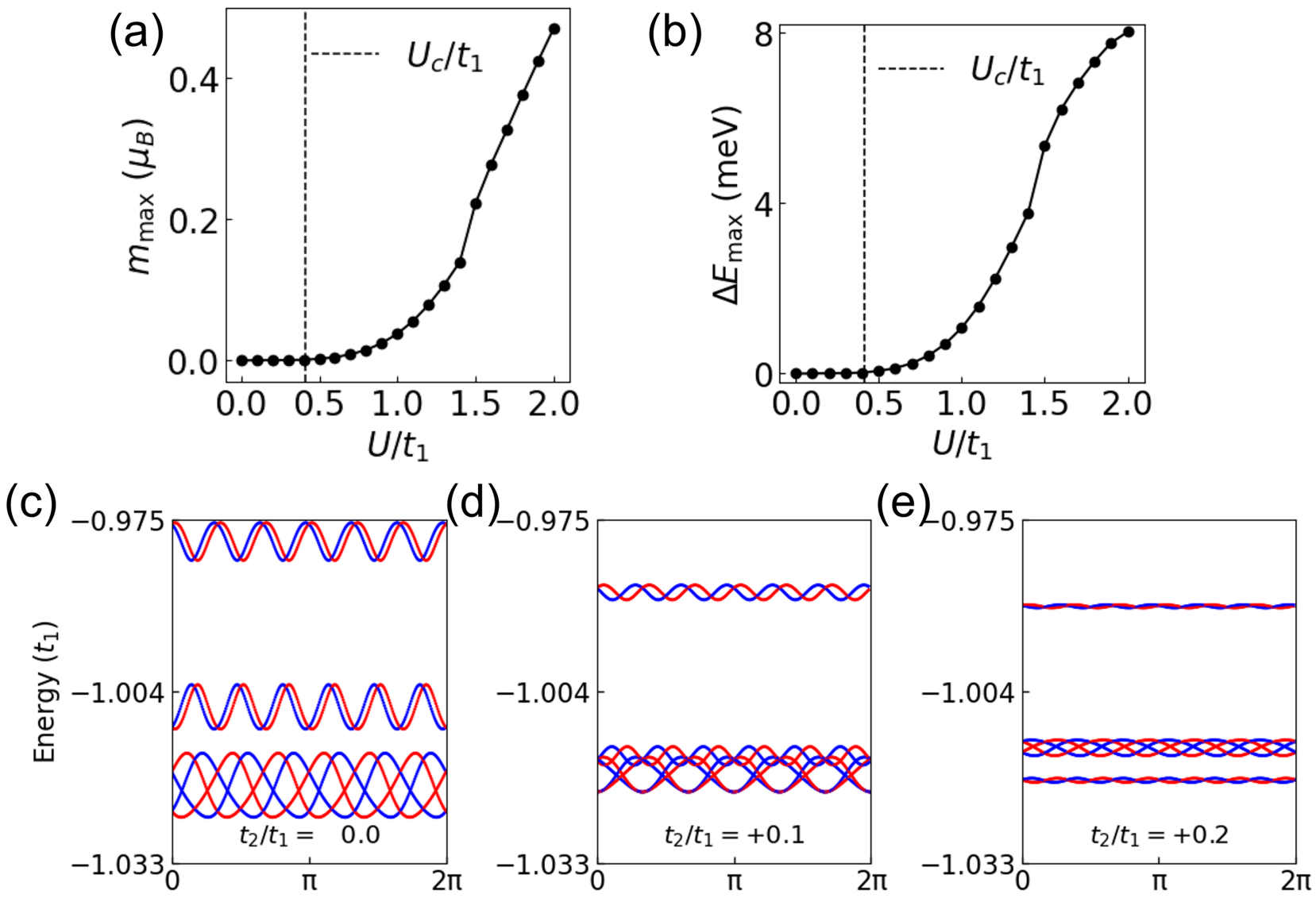


**Figure S1:** For monolayer graphene superlattice, (a) maximum local magnetic moment per C site, $m_{\max}$, as a function of $U/t_1$. (b) Maximum spin-splitting energy, $\Delta E_{\max}$, as a function of $U/t_1$, evaluated along the same circular $\Gamma$-centered path. (c–e) Spin-resolved band structures for $t_2/t_1 = 0.0$, 0.1, and 0.2, respectively. Energies are given in units of $t_1$.

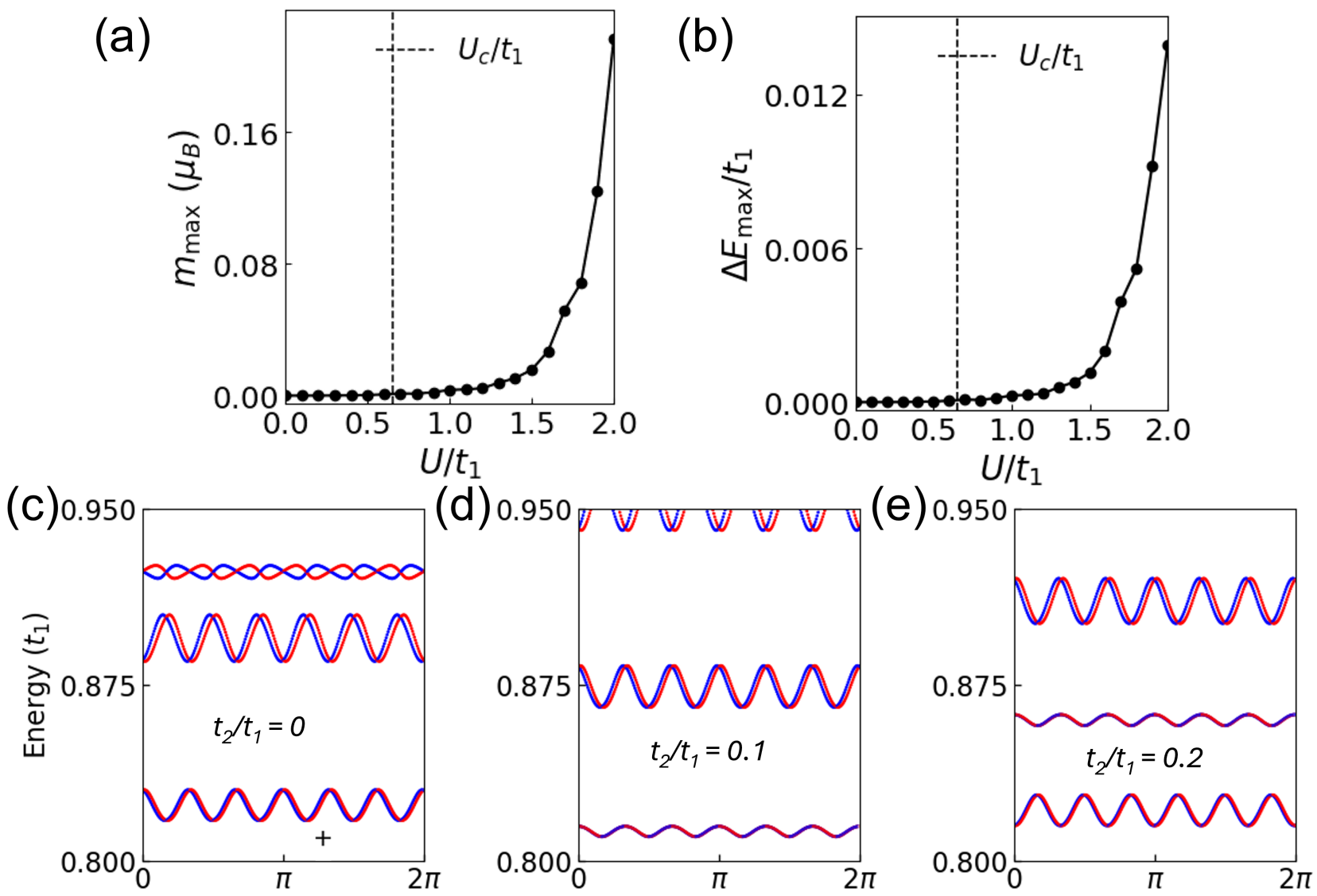


**Figure S2:** For AA bilayer graphene superlattice, (a) maximum local magnetic moment per C site, $m_{\max}$, as a function of $U/t_1$. (b) Maximum spin-splitting energy as a function of $U/t_1$. (c–e) Spin-resolved band structures for $t_2/t_1 = 0.0$, 0.1, and 0.2, respectively.

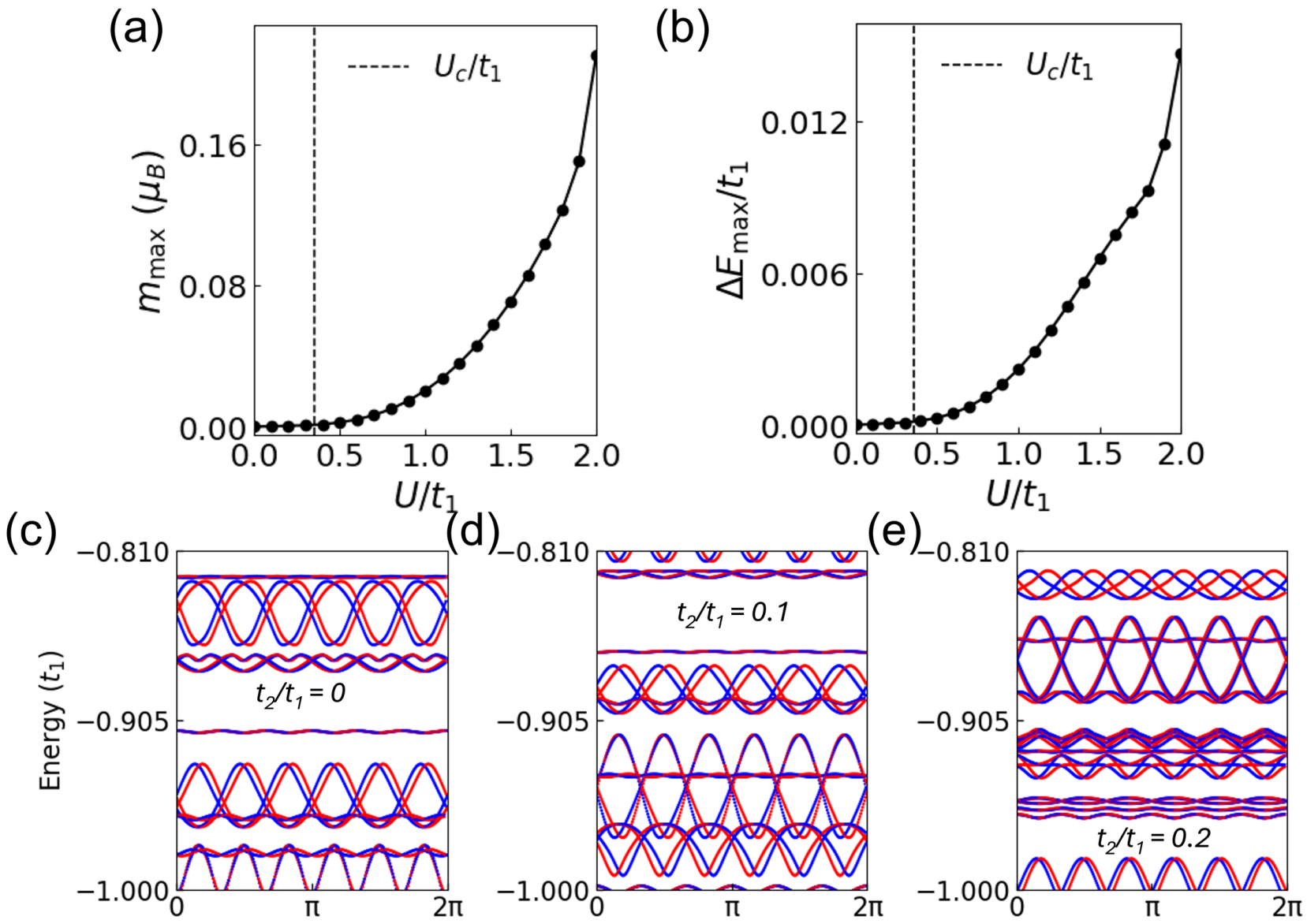


**Figure S3:** For AB bilayer graphene superlattice, (a) maximum local magnetic moment per C site, $m_{\rm max}$, as a function of $U/t_1$. (b) Maximum spin-splitting energy as a function of $U/t_1$. (c–e) Spin-resolved band structures for $t_2/t_1 = 0.0$, 0.1, and 0.2, respectively.

**Table S2:** Maximum spin splitting obtained from the model and from first-principles calculations for three representative systems. The model values are maxima over the full Brillouin zone.

| System | $\Delta E^{\rm model}_{\rm max}$ (meV) | $\Delta E^{\rm DFT}_{\rm max}$ (meV) |
|---|---|---|
| Monolayer | 34 | 33 |
| AA-stacked | 74 | 78 |
| AB-stacked | 50 | 45 |

# S4 Angular dependence of the spin splitting

We analyze the signed splitting of the band $n$ closest to the Fermi level, defined as

$$\Delta E_n(k_r, \theta) = E_{n\uparrow}(k_r, \theta) - E_{n\downarrow}(k_r, \theta), \tag{S6}$$

and decompose it as

$$\Delta E_n(k_r, \theta) = a_0 + \sum_{m=2,4,6} \left[a_m \cos(m\theta) + b_m \sin(m\theta)\right], \tag{S7}$$

where $A_m = \sqrt{a_m^2 + b_m^2}$ is the amplitude of the $m$-th harmonic. This selection rule is exact. With real hoppings and negligible spin-orbit coupling the splitting satisfies $\Delta E_n(\theta + 180^\circ) = \Delta E_n(\theta)$, while the $C_3$ symmetry of the antidot lattice imposes $\Delta E_n(\theta + 120^\circ) = \Delta E_n(\theta)$. Taken together these give a 60° period, so every harmonic below $m = 6$ vanishes identically rather than merely being small.

As shown in Fig. S4a-c, the $m = 2$ and $m = 4$ components vanish to numerical precision, staying below $10^{-11}$ meV (Fig. S4d), whereas the $m = 6$ component dominates at every

radius examined ($k_r = 0.3$, $0.4$, $0.5$). The vanishing angular average $a_0$ further excludes a uniform ferromagnetic-like splitting. The signed splitting also satisfies

$$\Delta E_n(\theta + \pi/6) \simeq -\Delta E_n(\theta), \tag{S8}$$

resulting in the twelve sign-changing sectors of an $i$-wave spin texture, and both the harmonic amplitudes and the normalized weights show that the $m = 6$ component dominates (Fig. S4d,e).

Fig. S4f shows a two-dimensional map of $E_{n\uparrow}(\mathbf{k}) - E_{n\downarrow}(\mathbf{k})$ across the first Brillouin zone, from which the angular profiles at $k_r = 0.3$, $0.4$ and $0.5$ are extracted. The same sign pattern and dominant $m = 6$ component persist at all radii, so the result is not specific to a single momentum-space contour.

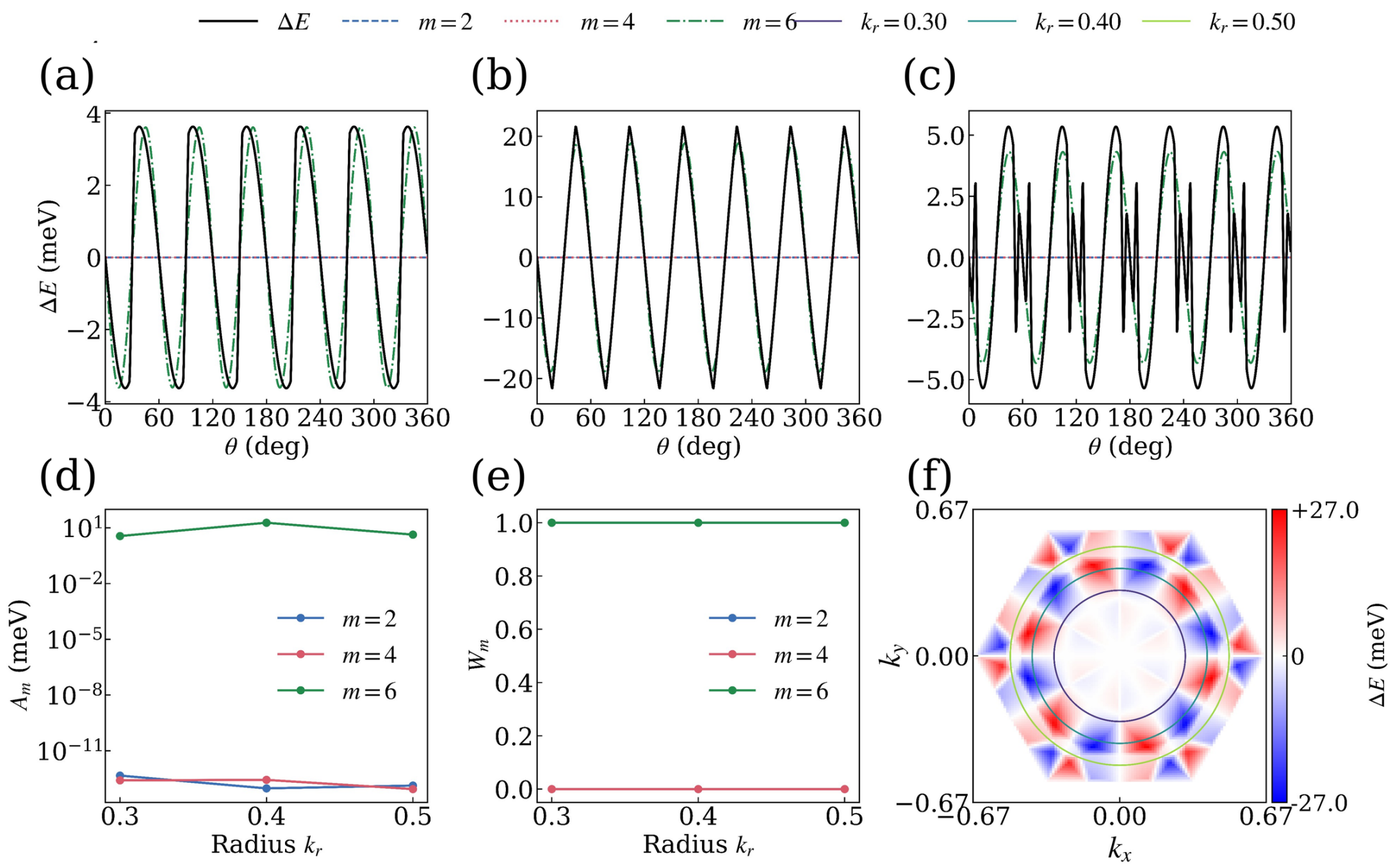


**Figure S4:** (a–c) Angular dependence of $\Delta E_n(k_r, \theta) = E_{n\uparrow}(k_r, \theta) - E_{n\downarrow}(k_r, \theta)$ for the fixed band closest to the Fermi level in monolayer graphene, evaluated at $k_r = 0.30$, $0.40$, and $0.50$, respectively. The black curves show the calculated splitting, while the dashed, dotted, and dash-dotted curves denote the $m = 2$, $m = 4$, and $m = 6$ harmonic components. (d,e) Radial dependence of the harmonic amplitudes $A_m$ and normalized weights $W_m$, demonstrating that the $m = 2$ and $m = 4$ contributions are negligible compared with the dominant $m = 6$ component at all examined radii. (f) Two-dimensional map of the signed spin splitting $\Delta E_n(\mathbf{k})$ in the first Brillouin zone. The colored circles indicate the momentum-space contours used in panels (a–e). The alternating sign pattern and robust $m = 6$ dominance across different radii establish the characteristic $i$-wave spin-splitting symmetry.

# S5 RPA spin response and spin stiffness

In the two-dimensional isotropic limit with negligible spin-orbit coupling, the Mermin-Wagner theorem rules out true long-range antiferromagnetic order at finite temperature, so a conventional Néel temperature is not well defined within these models [9]. Exchange interactions nevertheless sustain short-range antiferromagnetic correlations over a finite

temperature range, and we therefore estimate the spin stiffness as the relevant magnetic energy scale.

To estimate the characteristic magnetic energy scale, we calculate the transverse spin susceptibility within the random-phase approximation (RPA) and extract the low-energy acoustic magnon branch from the resulting spin excitation spectrum (Fig. S5). A gapless magnon branch appears at the Γ point, as required by the Goldstone theorem [10]. Near the Γ point the magnon dispersion is linear,

$$\frac{\omega}{t} \simeq vq, \tag{S9}$$

where $q$ is measured in units of the superlattice reciprocal vector $\mathbf{b}_1$. We obtain $v = 0.198$, 0.647, and 0.323 for the monolayer, AA-stacked bilayer, and AB-stacked bilayer, respectively. For the monolayer, this corresponds to $\hbar v = 1.88$ eV Å. The velocity is a direct output of the calculation and requires no modeling.

The quantity that controls the ordering temperature is not an exchange constant but the spin stiffness $\rho_s$, the energy cost of a long-wavelength twist of the Néel vector. We obtain it by mapping the RPA magnon onto linear spin-wave theory on the real-space nearest-neighbor network of the superlattice,

$$\rho_s = \frac{J}{A_{\text{cell}}} \sum_{\langle ij \rangle} S_i S_j \, (\hat{q} \cdot \boldsymbol{\delta}_{ij})^2, \tag{S10}$$

where $S_i$ is the converged local moment on site $i$, $\boldsymbol{\delta}_{ij}$ is the bond vector, $A_{\text{cell}}$ is the area of the magnetic unit cell, and $J$ is the overall energy scale of the mapping, fixed by matching the Heisenberg magnon slope to the RPA velocity. We evaluate the stiffness with the computed site-resolved moments rather than with a nominal $S = 1/2$.

This gives spin stiffnesses of $\rho_s \approx 177$ meV for the monolayer, $\rho_s \approx 158$ meV for the AA-stacked bilayer and $\rho_s \approx 159$ meV for the AB-stacked bilayer. Although the magnon velocities differ substantially, the stiffnesses remain comparable because the steeper branches of the bilayers are compensated by their smaller local magnetic moments. All values are approximately six to seven times larger than the room-temperature thermal energy scale of $k_\text{B}T \approx 25$ meV. Hence, the antiferromagnetic correlation length stays large at room temperature even though the isotropic two-dimensional limit forbids true long-range order. Since the magnetization in the vacancy superlattices is spatially nonuniform, the stiffness rather than a bond-resolved exchange constant is the meaningful characterization of these systems.

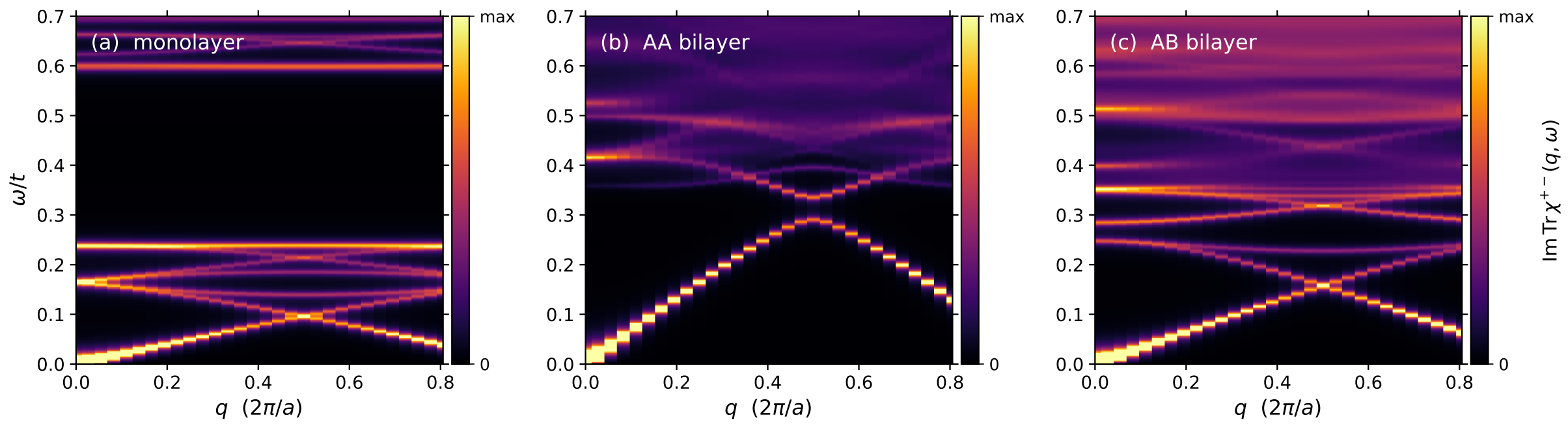


**Figure S5:** RPA transverse spin susceptibility $\chi''(q,\omega)$ for (a) the monolayer, (b) the AA-stacked bilayer, and (c) the AB-stacked bilayer graphene superlattices. The color scale is linear in $\mathrm{Im}\,\mathrm{Tr}\chi^{+-}$ and is clipped at the 99th percentile of each panel. The gapless branch emerging from $q=0$ is the acoustic magnon, whose slope gives the spin-wave velocity. All three panels share one grid, with $n_k=36$ and 30 momenta $q=n/36$.

# S6 Local-moment formation temperature

To complement the RPA spin-stiffness analysis we have performed finite-temperature mean-field calculations for the three structures, raising the temperature in the self-consistent Hubbard mean field until the magnetic solution collapses. The order parameter is the site-averaged local moment $\langle|m_z|\rangle$. The net moment vanishes at all temperatures because the state is a compensated antiferromagnet. The collapse temperature $T_{\mathrm{loc}}$ is obtained by extrapolating $\langle|m_z|\rangle^2$ to zero, which is linear in $T$ near the transition within mean-field theory. This gives $k_B T_{\mathrm{loc}} = 512$, 318 and 299 meV, that is 5940, 3690 and 3470 K, for the monolayer, the AA-stacked bilayer and the AB-stacked bilayer (Fig. S6).

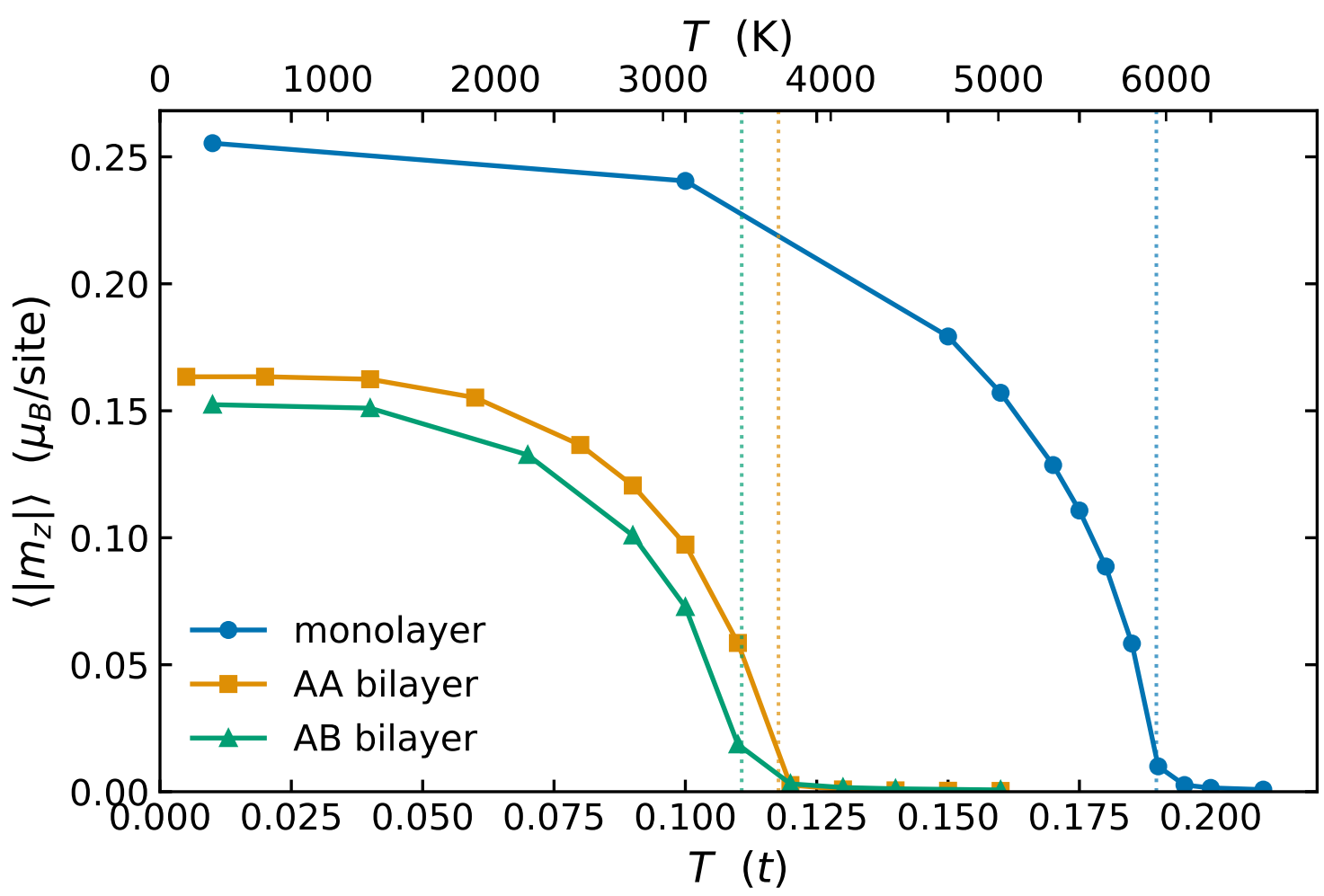


**Figure S6:** Site-averaged local moment $\langle|m_z|\rangle$ as a function of temperature, from finite-temperature mean-field theory at $U=2t$. The moments vanish at $T_{\mathrm{loc}}$ (dotted lines), obtained by extrapolating $\langle|m_z|\rangle^2$ to zero. This is the temperature at which the local moments cease to exist, and not a Néel temperature, since long-range order is absent at any finite temperature in the isotropic two-dimensional limit.

The two temperature scales measure different things. The mean-field calculation locates the temperature at which the local moments themselves disappear. Above $T_{\mathrm{loc}}$

the self-consistent solution no longer supports a finite $\langle |m_z| \rangle$ on any site, so there is no magnetism left to order. It is a local-moment formation scale, not an ordering temperature, and mean-field theory overestimates it because it omits the fluctuations that dominate a two-dimensional magnet. The onset of long-range order is instead controlled by the spin stiffness extracted from the RPA magnon.

The Mermin-Wagner theorem forbids long-range order only in the strictly isotropic limit. Spin-orbit coupling in these carbon systems is weak but finite, and the resulting magnetic anisotropy opens a gap in the magnon spectrum which cuts off the logarithmic infrared divergence of the thermal magnon population that would otherwise destroy the order. Because that cutoff enters only inside a logarithm, the ordering temperature is set by the spin stiffness and is only weakly sensitive to the size of the anisotropy, so even the small values computed here, 0.017 meV per C site for the monolayer and 0.006 meV per C site for the bilayers, are sufficient to stabilize order at a finite temperature. This is the same mechanism that gives monolayer $CrI_3$ a Curie temperature of 45 K despite its two-dimensionality [11].

This allows a direct estimate. Balancing the thermal magnon population against the ordered moment gives an ordering temperature of the form

$$k_B T_N \sim \frac{\rho_s}{\ln(\rho_s/\Delta)}, \tag{S11}$$

with $\Delta$ the anisotropy gap and a prefactor of order unity. Taking the measured intrinsic spin-orbit gap of graphene, $\Delta \approx 42$ $\mu$eV [12], together with the stiffnesses obtained above gives $\ln(\rho_s/\Delta) \approx 8$, and hence $k_B T_N \approx 21$ meV (245 K) for the monolayer and $k_B T_N \approx 19$ meV (225 K) for the AA- and AB-stacked bilayers. The estimate is only weakly sensitive to the anisotropy, which is its least certain input. Replacing $\Delta = 42$ $\mu$eV by the anisotropies computed here from first principles, 17 $\mu$eV and 6 $\mu$eV per C site, changes the logarithm by at most 24% and moves $T_N$ to 220 and 180 K. A sevenfold uncertainty in the anisotropy therefore costs less than a factor 1.3 in the ordering temperature, because the anisotropy enters only inside the logarithm while the stiffness sets the scale. These systems therefore order at a temperature of the order of room temperature, although no particular value should be taken as exact.

The mean field thus bounds the magnetic energy scale from above and the RPA stiffness sets the ordering scale below it, the former twelve to twenty times and the latter six to seven times the room-temperature thermal energy.

# S7 Stability verification

We examined the thermal and dynamical stability of the three structures through ab initio molecular dynamics (AIMD) simulations at 300 K and phonon calculations, respectively. During the 8 ps of simulation the structures remain intact, without bond breaking or noticeable structural reconstruction (Fig. S7). The calculated phonon spectra show no imaginary modes throughout the Brillouin zone (Fig. S8). The three systems are therefore stable against both finite-temperature fluctuations and small lattice perturbations.

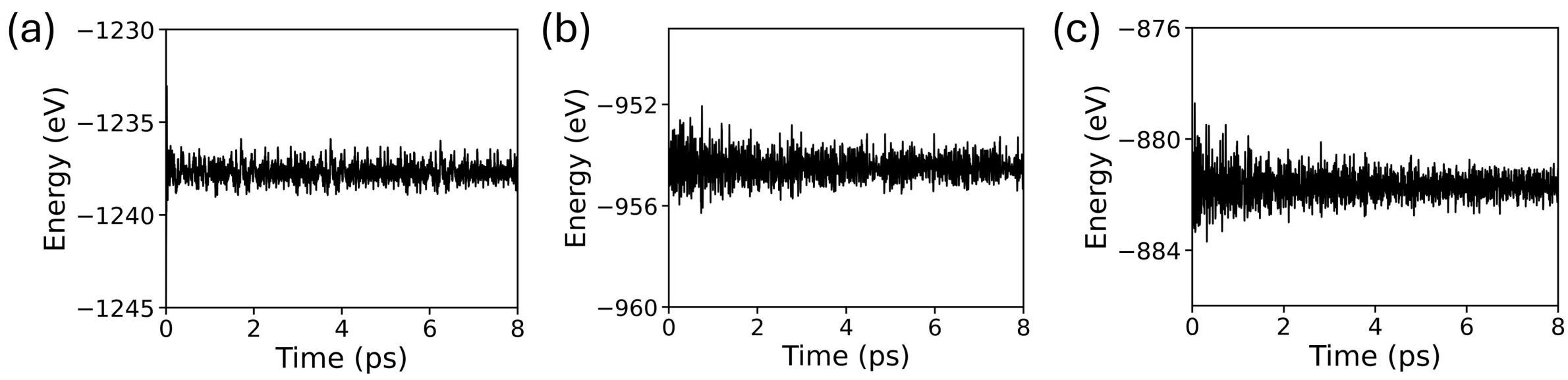


**Figure S7:** Total energy evolution during ab initio molecular dynamics (AIMD) simulations at 300 K over 8 ps for (a) the monolayer, (b) the AA-stacked bilayer and (c) the AB-stacked bilayer.

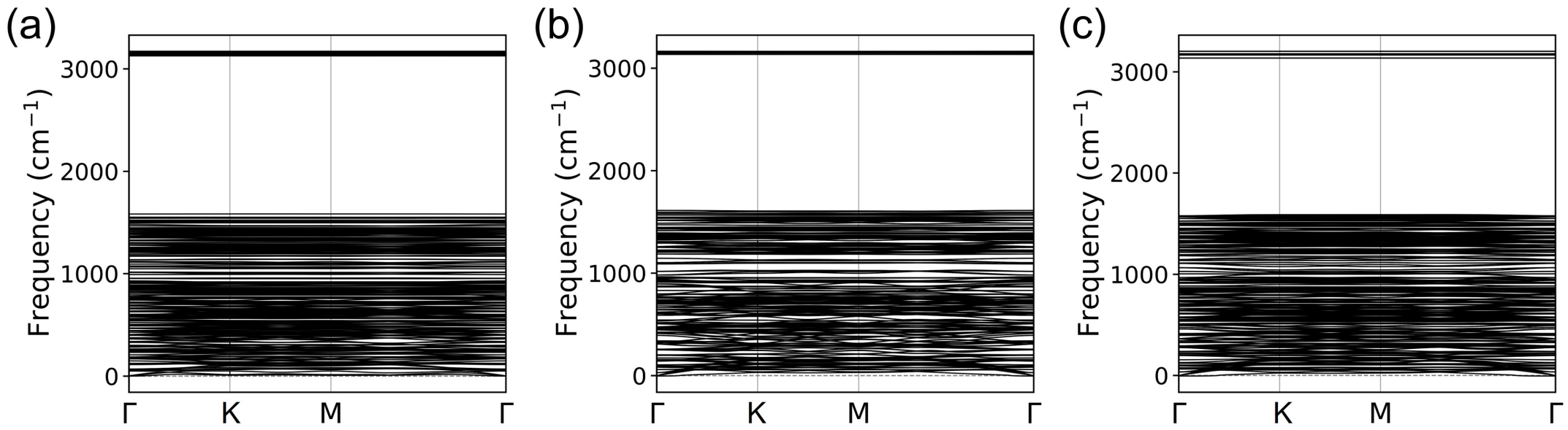


**Figure S8:** Phonon dispersions of the (a) monolayer, (b) AA-stacked bilayer, and (c) AB-stacked bilayer structures along the high-symmetry path.

# S8 Orbital-projected density of states

Using monolayer graphene as an example, we further identify the atomic orbitals contributing to the spin-split bands. The projected-density of states (DOS) analysis shows that these states are dominated by C $p_z$ orbitals, with minor contributions from C $p_x$ and $p_y$ orbitals, while the contribution from C $s$ orbitals is negligible (Fig. S9a). As shown in Fig. S9b, these states are distributed across the lattice rather than localized at the antidot edges.

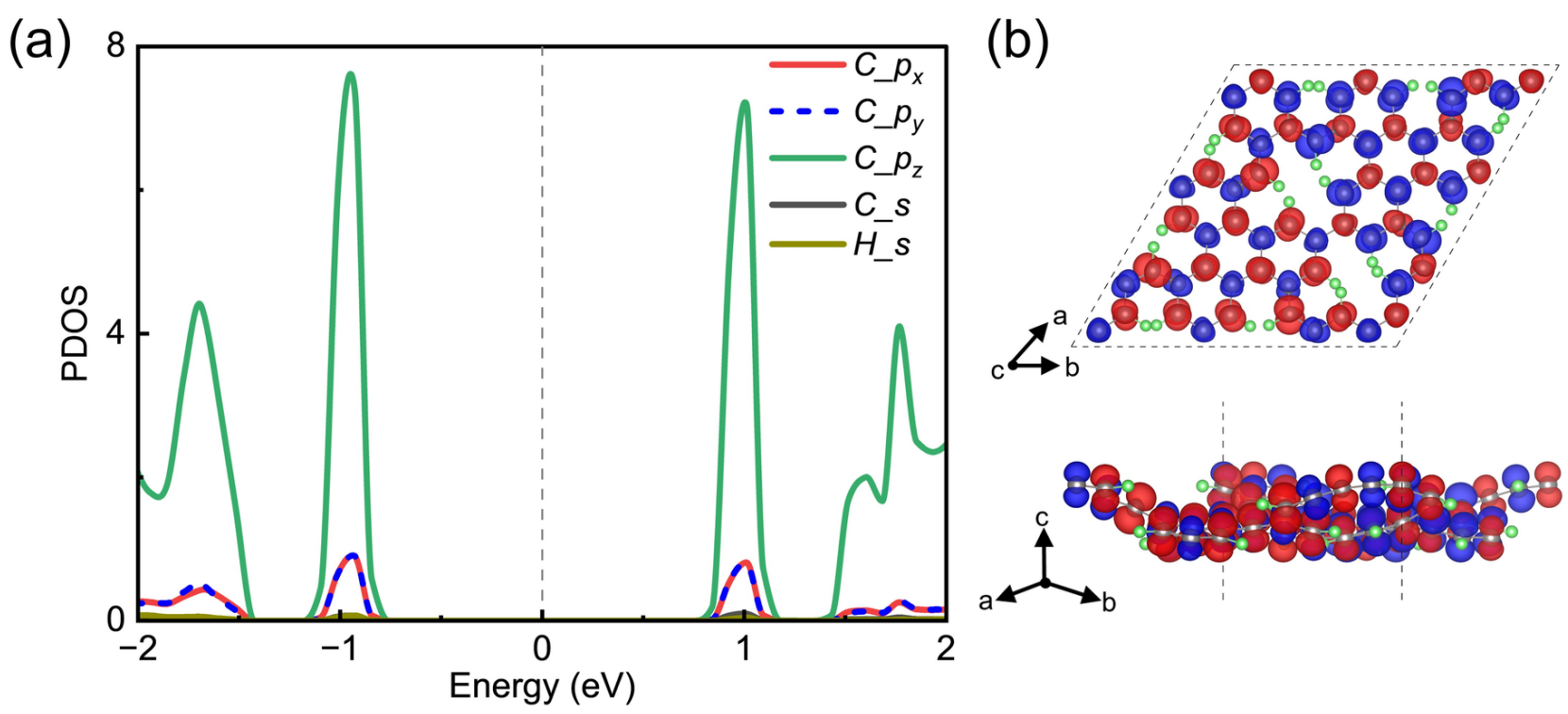


**Figure S9:** (a) Orbital-projected density of states of the monolayer antidot superlattice. (b) Top and side views of spin density distribution in the ground state, where red and blue denote spin-up and spin-down densities.

# S9 Comparison of planar and nonplanar monolayer band structures

We relaxed the monolayer superlattice under a planar constraint, optimizing the in-plane atomic coordinates while holding the out-of-plane positions fixed, and computed its band structure (Fig. S10a,b). The $i$-wave altermagnetic pattern of the planar structure is unchanged and consistent with the tight-binding model, and its spin splitting is comparable to that of the fully relaxed geometry (Fig. S10c,d). The structural buckling is therefore a symmetry-allowed distortion and not a prerequisite for altermagnetism.

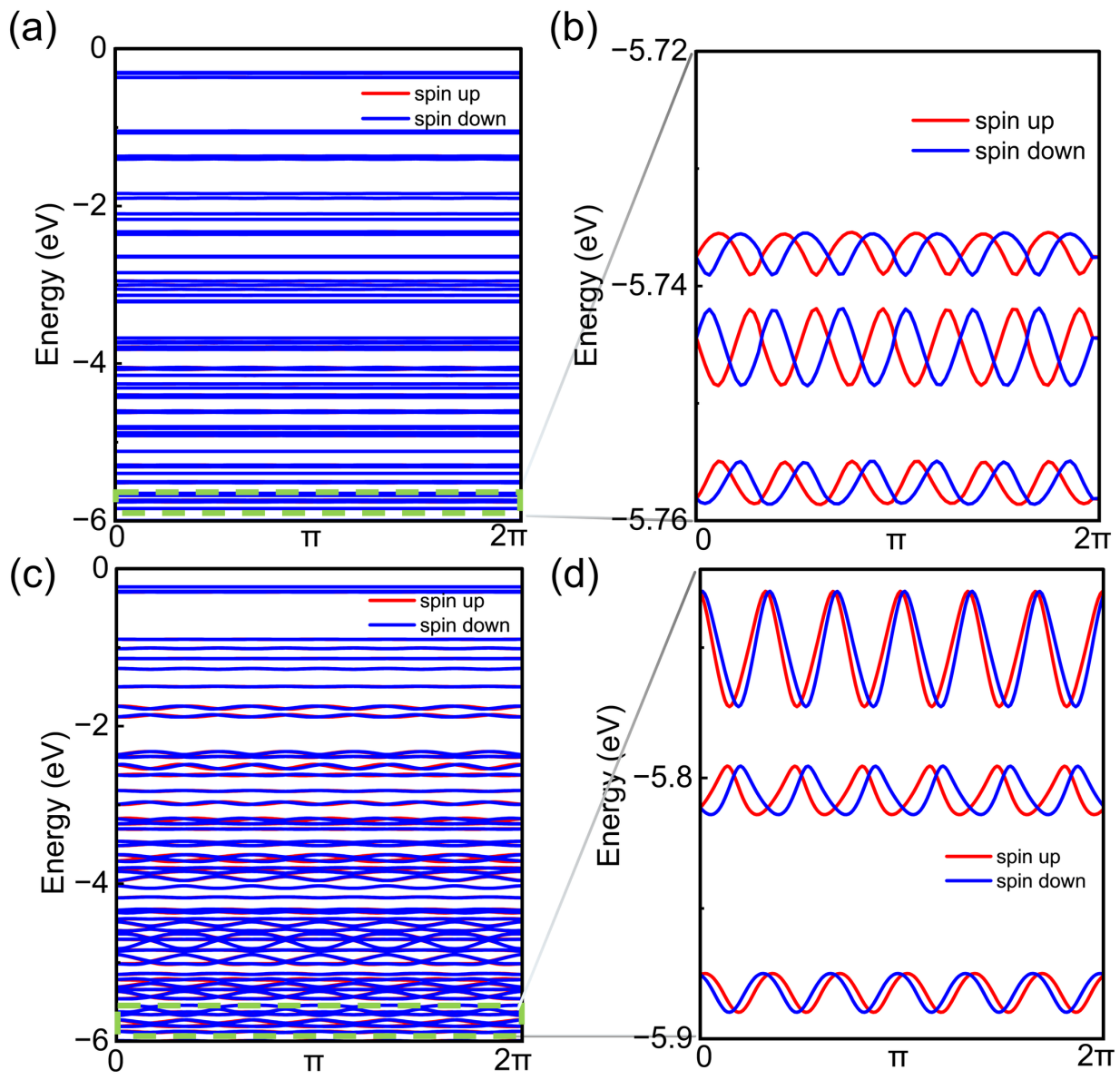


**Figure S10:** Band structures of monolayer graphene for (a,b) the planar structure, and (c,d) the fully relaxed nonplanar structure. Panels (b) and (d) show enlarged views of the regions highlighted by the green boxes in panels (a) and (c), respectively.

# S10 Nodal degeneracies in the fully relaxed structures

To quantify the effect of buckling on the nodal degeneracies, we evaluated the angular spin splitting $\Delta E(\theta) = E_{\uparrow}(\theta) - E_{\downarrow}(\theta)$ along a closed momentum-space path for the fully relaxed structures. As shown in Fig. S11, $\Delta E(\theta)$ changes sign through 12 nodal directions in the monolayer, AA-stacked bilayer, and AB-stacked bilayer, with the interpolated splitting vanishing at each crossing within numerical resolution. Buckling therefore modifies the magnitude and angular distribution of the spin splitting rather than lifting the nodal degeneracies.

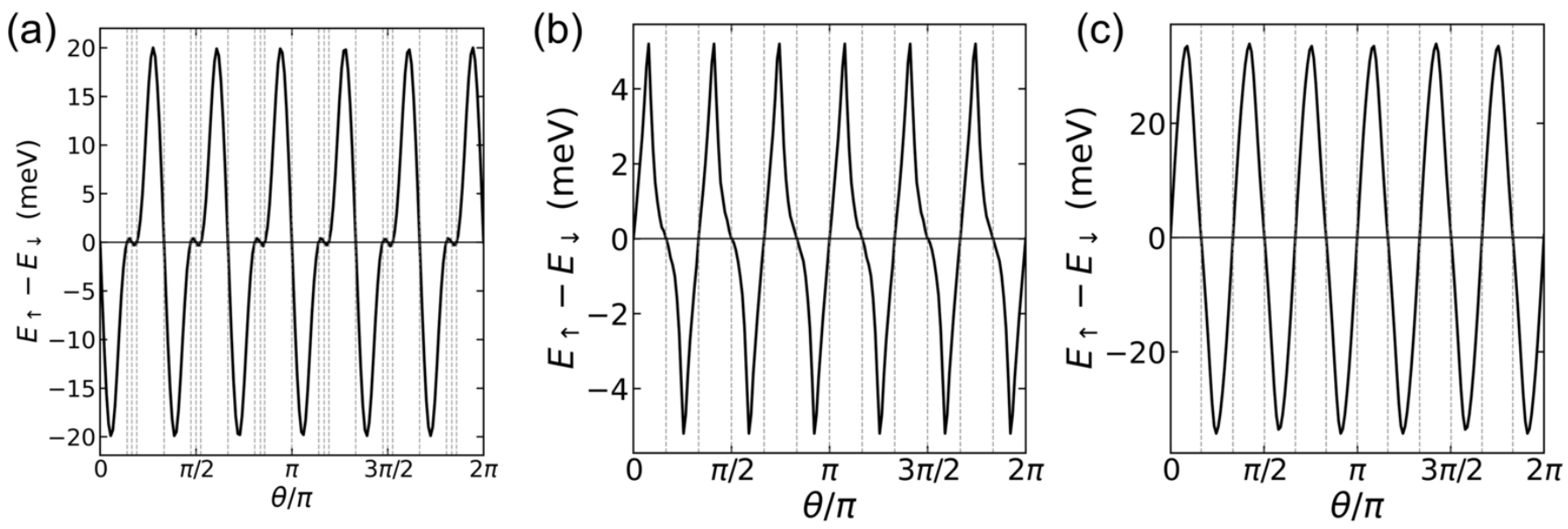


**Figure S11:** Angular spin splitting $\Delta E(\theta) = E_\uparrow - E_\downarrow$ for the fully relaxed (a) monolayer, (b) AA-stacked bilayer, and (c) AB-stacked bilayer structures. The gray dashed lines mark the 12 sign-changing nodal crossings. Here, the split band is chosen from the occupied state near the Fermi level.

# S11 Spin-resolved band structures along different $k$-paths

Fig. S12a–c shows the first-principles spin-resolved bands on a circular path whose radius is chosen so that the states within 0.6 eV of the Fermi level carry a resolvable $i$-wave splitting. In the first-principles band structures the Fermi level is placed at the valence-band maximum, so this depth is measured from the band edge. We also calculated the band structures along the conventional high-symmetry $k$-path, as shown in Fig. S12d–f. The twelve nodal directions of an $l = 6$ form factor are spaced by 30°, and in a hexagonal Brillouin zone the Γ–M and Γ–K directions are separated by exactly that angle, so both of them are nodal. Both radial segments of the conventional path therefore carry no altermagnetic splitting by symmetry, and only the zone-boundary segment samples off-nodal directions. This is why the bands are displayed on a circular path of fixed $|k|$ in panels a–c, which sweeps the angle and exposes the sixfold sign alternation directly. In addition, varying the antidot size or spacing in graphene can tune the defect-state hybridization, magnetic moment, and band gap, and may shift the spin-split features closer to the Fermi level [13].

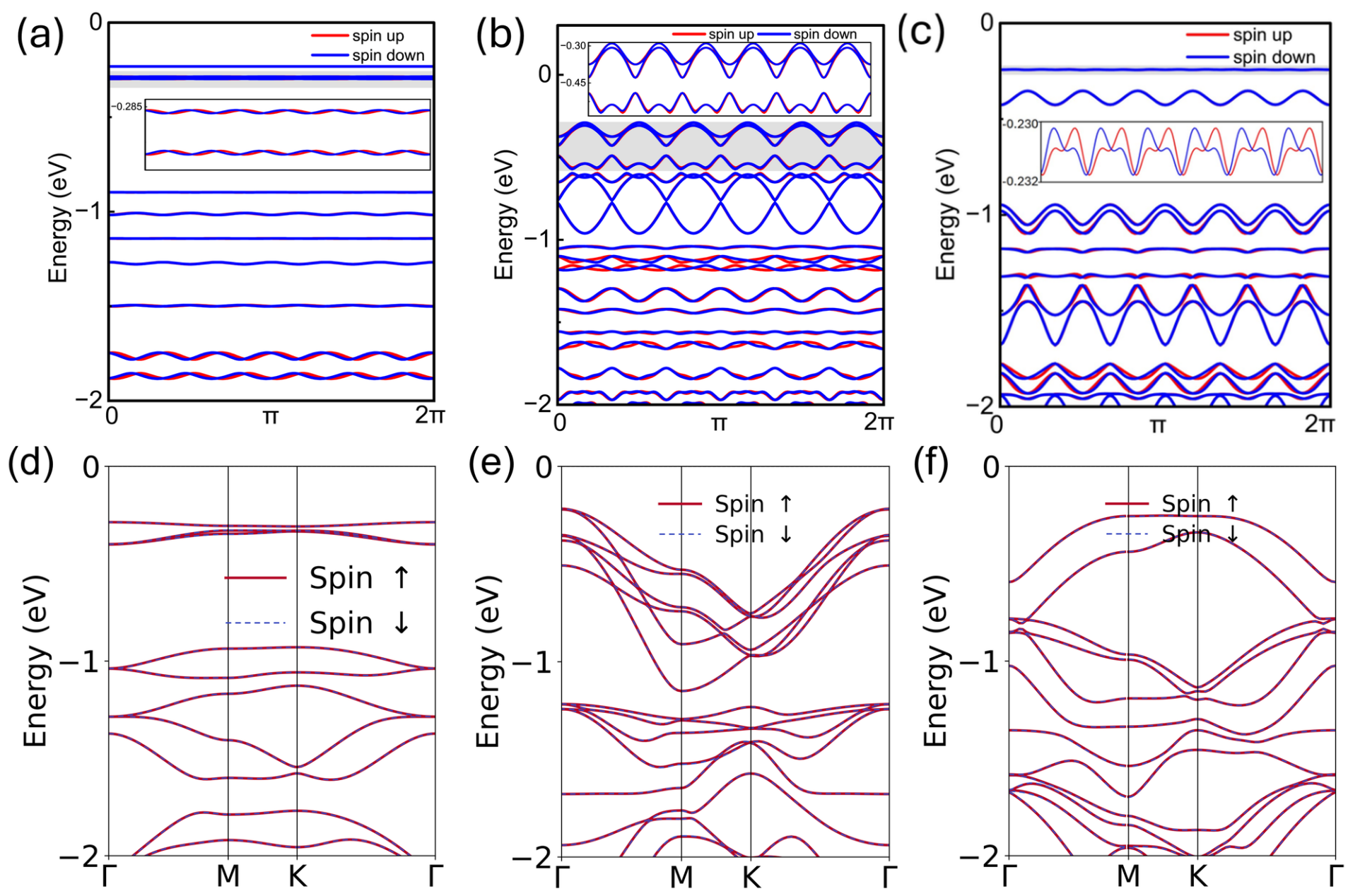


**Figure S12:** Spin-resolved band structures along (a–c) a circular $k$-path and (d–f) the conventional linear $k$-path, with enlarged views of the gray-shaded regions near the Fermi level, for (a,d) the monolayer graphene superlattice, (b,e) the AA bilayer and (c,f) the AB bilayer. The insets show the first Brillouin zone and the selected momentum-space paths.

We also quantify the doping requirement by the chemical-potential shift from charge neutrality needed to reach the spin-split states. In the tight-binding model, splittings of $\sim 10$ meV occur at $0.35t$ and $0.65t$ for the AA and AB bilayers, respectively, while the monolayer requires a larger shift of $0.83t$ to reach 8 meV. DFT shows the same trend, with weak near Fermi level splittings of 1.6 to 8.1 meV and larger splittings of 16 to 18 meV at deeper energies (Fig. S12a–c). Because such large shifts may be difficult to achieve by gating, spin-ARPES provides an alternative probe of the deeper spin-split states (see Sec. S13 for more details). A doping of this size would in any case quench the altermagnetic moment itself. The moments here form on the half-filled midgap states of the antidot lattice, which is the same mechanism that produces defect-induced magnetism in graphene [14–16], and charging those states removes the local moment that the splitting is built on [17].

# S12 Spin-orbit coupling and magnetic anisotropy

To evaluate the SOC induced by the structural distortion, we performed fully relativistic calculations including SOC for the fully relaxed geometries. The magnetic anisotropy energy between the Néel-vector orientations along [001] and [100] is 0.017 meV per C site for the monolayer and 0.006 meV per C site for the bilayers, a negligible SOC-induced magnetic anisotropy. The intrinsic spin-orbit coupling of flat graphene is itself only 24 $\mu$eV and, as first-principles calculations show, comes mainly from the $d$ orbitals [18], so the anisotropies computed here for the buckled superlattices are of the same order.

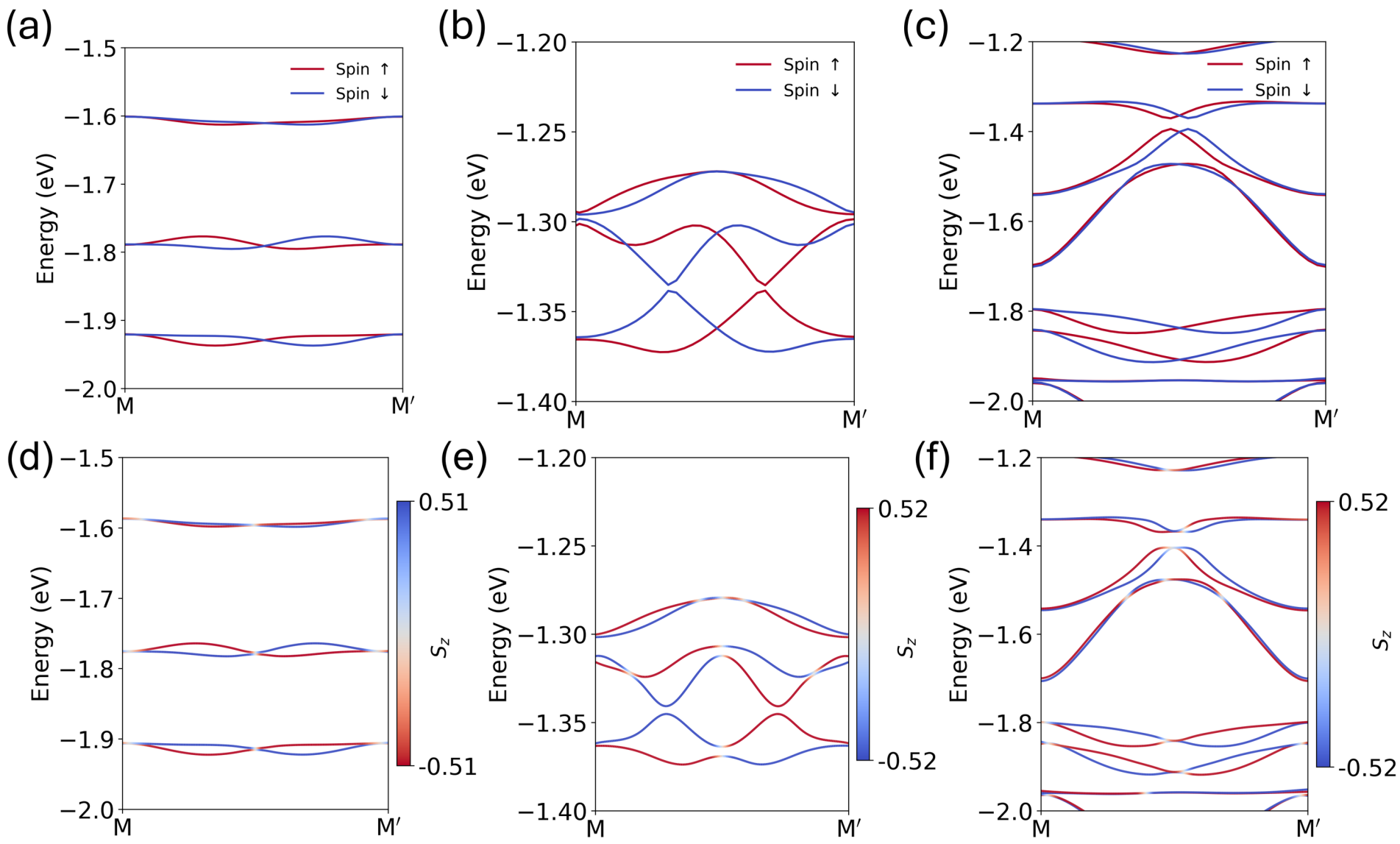


**Figure S13:** Spin-resolved band structures of the graphene superlattice without SOC (a–c) and with SOC (d–f) for the monolayer, AA bilayer, and AB bilayer, respectively. Here, M = (0.5, 0, 0) and M' = (0, 0.5, 0).

As displayed in Fig. S13, the band structure with SOC remains nearly indistinguishable from that without SOC, and the characteristic momentum-dependent spin splitting is preserved. Therefore, although SOC formally reduces the nonrelativistic spin-space symmetry, the deformation-induced SOC is a weak relativistic perturbation, three orders of magnitude below the exchange-driven altermagnetic splitting, and does not destabilize the $i$-wave altermagnetism.

# S13 Magnonic detection of the $i$-wave order

The graphene superlattices studied here are magnetic insulators, with a tight-binding gap of 1.96 eV for the monolayer and 0.99 eV for both bilayers and the chemical potential in the gap. This rules out the charge-transport fingerprints usually invoked for altermagnets. With the chemical potential in the gap the occupation factor is unity on every valence band and zero on every conduction band, so any Drude-type response reduces to the Brillouin-zone integral of a momentum derivative of a smooth periodic function, which vanishes identically at every order in the driving field. The absence of a low-order transport response is then a property shared by any band insulator and carries no information about the wave symmetry.

There is a further, purely symmetry-based obstruction. An $i$-wave form factor is an $l = 6$ angular harmonic, whereas a response tensor of rank $r$ carries angular components only up to $m = r$. Any Brillouin-zone-integrated response of rank five or lower is therefore blind to $i$-wave order regardless of the filling. Momentum-resolved probes are not subject to this constraint, and two such routes are described in the following.

First, spin-resolved angle-resolved photoemission spectroscopy (spin-ARPES) provides a direct probe of the electronic spin splitting in occupied bands lying several eV below the Fermi level [19]. It reconstructs the spin-resolved band dispersion from the energy, emission angle, and spin polarization of photoemitted electrons. Spin-resolved photoemission has resolved spin splittings of about 100 meV in the Dirac cone of graphene on Au [20]

and of up to 0.5 eV in $WSe_2$ [21], and photoemission with a micrometer-scale focus now maps the band structure of exfoliated flakes and devices, including twisted bilayer graphene [22], encapsulated few-layer $WTe_2$ [23] and gated graphene heterostructures [24]. The pronounced higher-energy splitting predicted in our systems should therefore be directly accessible by spin-ARPES, as demonstrated recently for altermagnetic MnTe [25] and CrSb [26].

Second, the magnon spectrum is the standard probe of altermagnetism in insulators. In a compensated collinear magnet the two magnon branches carry opposite spin chirality, and in an altermagnet they are split by an amount that follows the same angular form factor as the electronic spin splitting,

$$\omega_{+}(\mathbf{k}) - \omega_{-}(\mathbf{k}) \;\propto\; \Delta(\mathbf{k}). \tag{S12}$$

The magnon splitting therefore inherits the nodal structure of the band splitting. For the $i$-wave state realized here this gives a sixfold-periodic, sign-alternating magnon splitting with twelve nodal directions in $\mathbf{k}$, the same twelve directions identified in the electronic structure, now appearing in the magnon dispersion. Because the effect is nonrelativistic it does not require spin-orbit coupling, which is an advantage in a carbon-based platform, where a measured splitting cannot be attributed to relativistic effects.

We have computed this splitting directly. Evaluating the poles of the RPA kernel on a circle of fixed $|q|$ in the AA-stacked bilayer gives the magnon energy $\omega(\varphi)$ around the circle. That energy is dominated by the nonmagnetic hexagonal warping of the lattice, which is 25.3 meV peak to peak and would bury the effect of interest. The altermagnetic contribution is instead the part of $\omega(\varphi)$ that is odd under the mirror $\varphi \to 60^\circ - \varphi$. That mirror exchanges the two magnetic sublattices and is broken only by the magnetic order, whereas the warping is even under it and cancels exactly. This odd part is shown in Fig. S14. It is sixfold periodic with twelve nodes, carries 97% of its angular power in the $l = 6$ harmonic, and splits the two magnon branches by 0.56 meV. The agreement of that harmonic content with the electronic form factor confirms that the magnon splitting carries the same $i$-wave symmetry as the band splitting.

The radial path used for the $\chi''$ maps of Fig. S5 runs along $\mathbf{b}_1$, which is one of these twelve nodal directions, so those maps show no chiral splitting at any level of convergence. A circular path is needed to see it, which is why the splitting is presented as a function of angle rather than as a further panel of Fig. S5.

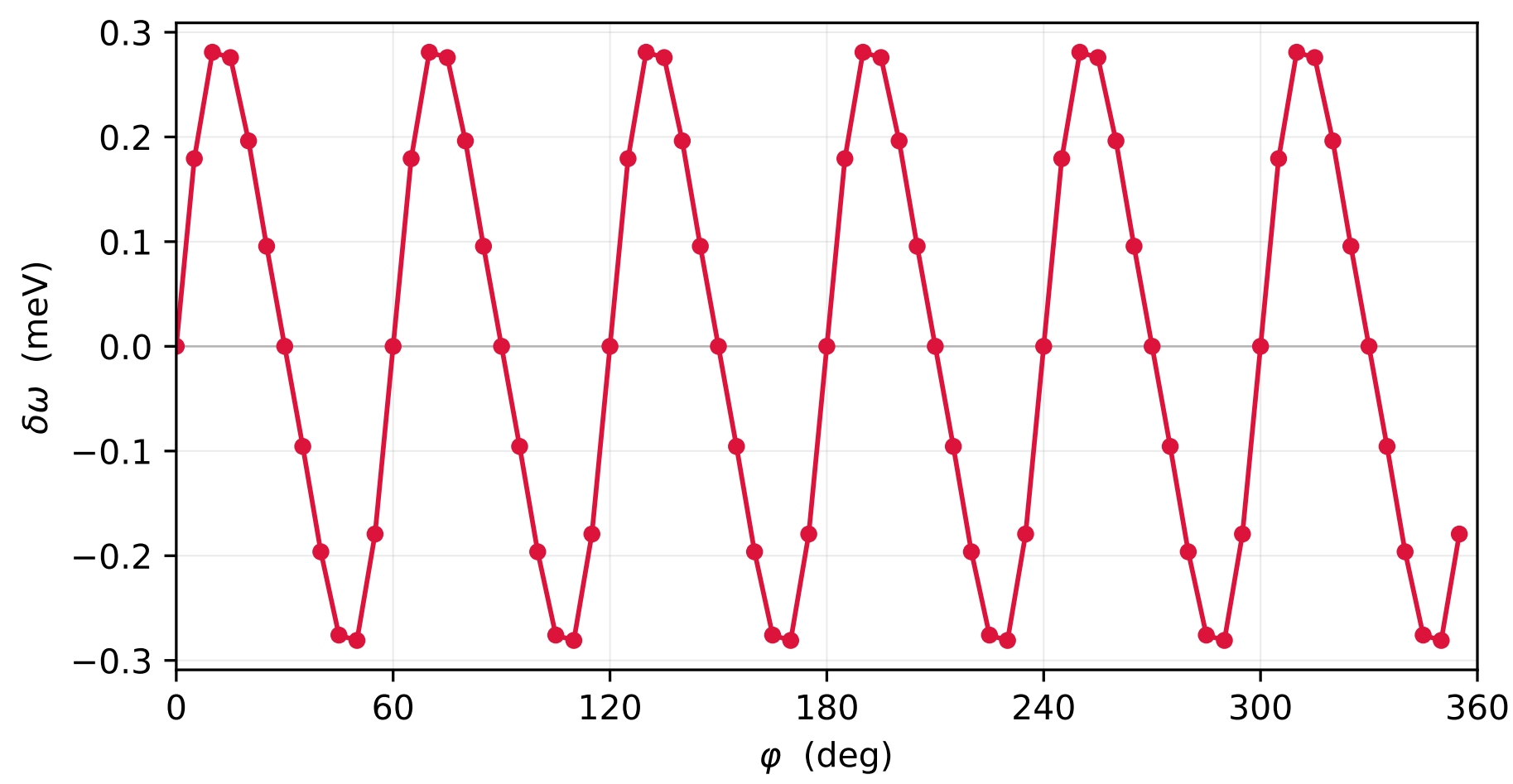


**Figure S14:** Chiral magnon splitting on a circular path of fixed $|q|$ in the AA-stacked bilayer. The plotted quantity is the part of the magnon energy that is odd under the mirror $\varphi \to 60^\circ - \varphi$, namely $\delta\omega(\varphi) = [\omega(\varphi) - \omega(60^\circ - \varphi)]/2$. That mirror exchanges the two magnetic sublattices, so the nonmagnetic hexagonal warping of the lattice, which is 25.3 meV peak to peak and would otherwise bury the effect, is even under it and cancels exactly. Every mirror partner is a computed point of the $5^\circ$ grid, so nothing is interpolated. Obtained from the poles of the RPA kernel at $n_k = 36$, with $|q| = 0.55 \times 2\pi/a$.

This signature is established in comparable materials. Chiral magnon splitting with alternating chirality was predicted from first principles for the $d$-wave altermagnet $RuO_2$ [27] and has been identified in bulk altermagnetic MnTe [28]. In a two-dimensional sample the magnon spectrum is reached through magnon spin transport. Long-distance magnon transport has been measured in atomically thin van der Waals antiferromagnets [29], and the spin-split magnon bands of an insulating altermagnet carry spin Seebeck and spin Nernst currents that require neither a magnetic field nor spin-orbit coupling [30], as shown for a collinear honeycomb geometry closely related to ours [31]. Noise magnetometry has been proposed as a way of separating altermagnetic from antiferromagnetic order through the magnon contribution alone, with no mobile carriers required [32]. The electronic spin splitting that sets the magnon form factor is in turn directly accessible to spin-ARPES, as described above.

The magnon splitting is robust against averaging over time-reversed altermagnetic domains, provided the domains are large compared with the length scale set by the momentum resolution of the probe. Each domain hosts the two branches at $\omega_0 \pm \Delta(\mathbf{k})$, and the time-reversed domain only exchanges which branch carries which spin chirality, so a domain average retains the splitting and its twelve nodal directions. What averaging cancels is the chirality-resolved sign of the splitting, together with the time-reversal-odd magnon spin Nernst and Seebeck signals, and recovering that sign requires a single-domain measurement or an unequal population of the two domains.

# S14 Supercell selection and symmetry preservation

The tight-binding calculations employ a $6\sqrt{3}\times6\sqrt{3}$ supercell for the monolayer, a $3\sqrt{3}\times3\sqrt{3}$ supercell for the AA-stacked bilayer and a $5\sqrt{3} \times 5\sqrt{3}$ supercell for the AB-stacked bilayer. The first-principles calculations use $6\times6$, $3\sqrt{3}\times3\sqrt{3}$, and $5\times5$ supercells, respectively. The

tight-binding cells follow the antidot superlattice construction, while for the first-principles calculations we adopt the smallest computationally affordable cells that preserve the same target spin point-group symmetry as the corresponding tight-binding models. We have verified that these first-principles structures retain the required symmetry and exhibit the same $i$-wave altermagnetic spin-splitting pattern. Although the tight-binding and first-principles calculations therefore correspond to different superlattice periodicities for the monolayer and the AB-stacked bilayer, they realize the same altermagnetic symmetry.

## S15 Feasibility of AA-stacked graphene

AA stacking is not the ground-state configuration of pristine bilayer graphene, but AA-stacked bilayer graphene is experimentally accessible. AA-stacked phases were identified in epitaxially grown bilayer graphene on Ru(0001) [33]. In addition, AA-stacked regions are directly imaged by scanning tunneling microscopy in marginally twisted bilayer graphene, where they carry a pronounced localized tunneling peak and can be tuned by strain [34]. A local AA environment is thus accessible even without globally AA-stacked material.

## References


[1] S. Reich, J. Maultzsch, C. Thomsen, and P. Ordejon. Tight-binding description of graphene. *Phys. Rev. B*, 66(3):035412, 2002.

[2] G. Trambly de Laissardière, D. Mayou, and L. Magaud. Numerical studies of confined states in rotated bilayers of graphene. *Phys. Rev. B*, 86(12):125413, 2012.

[3] J. L. Lado. Pyqula library. https://github.com/joselado/pyqula, 2026.

[4] M. Schüler, M. Rösner, T. Wehling, A. Lichtenstein, and M. Katsnelson. Optimal Hubbard models for materials with nonlocal Coulomb interactions: Graphene, silicene, and benzene. *Phys. Rev. Lett.*, 111(3):036601, 2013.

[5] C. Le, F. Cui, I.-F. Io, M. Hirschmann, X. Wu, and C.-K. Chiu. Catalog of altermagnetism in magnetic wallpaper/space groups and nonsymmorphic altermagnets. *arXiv*, 2026. arXiv:2607.10857.

[6] M. Naka, Y. Motome, and H. Seo. Altermagnetic perovskites. *npj Spintron.*, 3(1):1, 2025.

[7] L. Šmejkal, J. Sinova, and T. Jungwirth. Beyond conventional ferromagnetism and antiferromagnetism: A phase with nonrelativistic spin and crystal rotation symmetry. *Phys. Rev. X*, 12(3):031042, 2022.

[8] L. Šmejkal, J. Sinova, and T. Jungwirth. Emerging research landscape of altermagnetism. *Phys. Rev. X*, 12(4):040501, 2022.

[9] N. D. Mermin and H. Wagner. Absence of ferromagnetism or antiferromagnetism in one- or two-dimensional isotropic Heisenberg models. *Phys. Rev. Lett.*, 17(22):1133–1136, 1966.

[10] H. Wagner. Long-wavelength excitations and the Goldstone theorem in many-particle systems with "broken symmetries". *Zeitschrift für Physik*, 195(3):273–299, 1966.

[11] J. L. Lado and J. Fernández-Rossier. On the origin of magnetic anisotropy in two dimensional $CrI_3$. *2D Mater.*, 4(3):035002, 2017.

[12] J. Sichau, M. Prada, T. Anlauf, T. J. Lyon, B. Bosnjak, L. Tiemann, and R. H. Blick. Resonance microwave measurements of an intrinsic spin-orbit coupling gap in graphene: A possible indication of a topological state. *Phys. Rev. Lett.*, 122(4):046403, 2019.

[13] J. A. Fürst, T. G. Pedersen, M. Brandbyge, and A.-P. Jauho. Density functional study of graphene antidot lattices: Roles of geometrical relaxation and spin. *Phys. Rev. B*, 80(11):115117, 2009.

[14] O. V. Yazyev and L. Helm. Defect-induced magnetism in graphene. *Phys. Rev. B*, 75(12):125408, 2007.

[15] J. J. Palacios, J. Fernández-Rossier, and L. Brey. Vacancy-induced magnetism in graphene and graphene ribbons. *Phys. Rev. B*, 77:195428, 2008.

[16] O. V. Yazyev. Emergence of magnetism in graphene materials and nanostructures. *Rep. Prog. Phys.*, 73(5):056501, 2010.

[17] J. Fernández-Rossier and J. J. Palacios. Magnetism in graphene nanoislands. *Phys. Rev. Lett.*, 99(17):177204, 2007.

[18] M. Gmitra, S. Konschuh, C. Ertler, C. Ambrosch-Draxl, and J. Fabian. Band-structure topologies of graphene: Spin-orbit coupling effects from first principles. *Phys. Rev. B*, 80(23):235431, 2009.

[19] P. Ngabonziza, J. D. Denlinger, A. V. Fedorov, G. Cao, J. W. Allen, G. Gebreyesus, and R. M. Martin. Layer-dependent spin-resolved electronic structure of ferromagnetic triple-layered ruthenate $Sr_4Ru_3O_{10}$. *Phys. Rev. B*, 111(11):115146, 2025.

[20] D. Marchenko, A. Varykhalov, M. R. Scholz, G. Bihlmayer, E. I. Rashba, A. Rybkin, A. M. Shikin, and O. Rader. Giant Rashba splitting in graphene due to hybridization with gold. *Nat. Commun.*, 3(1):1232, 2012.

[21] J. M. Riley, F. Mazzola, M. Dendzik, M. Michiardi, T. Takayama, L. Bawden, C. Granerød, M. Leandersson, T. Balasubramanian, M. Hoesch, T. K. Kim, H. Takagi, W. Meevasana, P. Hofmann, M. S. Bahramy, J. W. Wells, and P. D. C. King. Direct observation of spin-polarized bulk bands in an inversion-symmetric semiconductor. *Nat. Phys.*, 10(11):835–839, 2014.

[22] S. Lisi, X. Lu, T. Benschop, T. A. de Jong, P. Stepanov, J. R. Duran, F. Margot, I. Cucchi, E. Cappelli, A. Hunter, A. Tamai, V. Kandyba, A. Giampietri, A. Barinov, J. Jobst, V. Stalman, M. Leeuwenhoek, K. Watanabe, T. Taniguchi, L. Rademaker, S. J. van der Molen, M. P. Allan, D. K. Efetov, and F. Baumberger. Observation of flat bands in twisted bilayer graphene. *Nat. Phys.*, 17(2):189–193, 2021.

[23] I. Cucchi, I. Gutiérrez-Lezama, E. Cappelli, S. McKeown Walker, F. Y. Bruno, G. Tenasini, L. Wang, N. Ubrig, C. Barreteau, E. Giannini, M. Gibertini, A. Tamai, A. F. Morpurgo, and F. Baumberger. Microfocus laser-angle-resolved photoemission on encapsulated mono-, bi-, and few-layer 1T′-$WTe_2$. *Nano Lett.*, 19(1):554–560, 2019.

[24] P. V. Nguyen, N. C. Teutsch, N. P. Wilson, J. Kahn, X. Xia, A. J. Graham, V. Kandyba, A. Giampietri, A. Barinov, G. C. Constantinescu, N. Yeung, N. D. M. Hine, X. Xu, D. H. Cobden, and N. R. Wilson. Visualizing electrostatic gating effects in two-dimensional heterostructures. *Nature*, 572(7768):220–223, 2019.

[25] J. Krempaský, L. Šmejkal, S. W. D'Souza, M. Hajlaoui, G. Springholz, K. Uhlířová, F. Alarab, P. C. Constantinou, V. Strocov, D. Usanov, W. R. Pudelko, R. González-Hernández, A. Birk Hellenes, Z. Jansa, H. Reichlová, Z. Šobáň, R. D. Gonzalez Betancourt, P. Wadley, J. Sinova, D. Kriegner, J. Minár, J. H. Dil, and T. Jungwirth. Altermagnetic lifting of Kramers spin degeneracy. *Nature*, 626(7999):517–522, 2024.

[26] G. Yang, Z. Li, S. Yang, J. Li, H. Zheng, W. Zhu, Z. Pan, Y. Xu, S. Cao, W. Zhao, A. Jana, J. Zhang, M. Ye, Y. Song, L.-H. Hu, L. Yang, J. Fujii, I. Vobornik, M. Shi, H. Yuan, Y. Zhang, Y. Xu, and Y. Liu. Three-dimensional mapping of the altermagnetic spin splitting in CrSb. *Nat. Commun.*, 16(1):1442, 2025.

[27] L. Šmejkal, A. Marmodoro, K. H. Ahn, R. González-Hernández, I. Turek, S. Mankovsky, H. Ebert, S. W. D'Souza, O. Šipr, J. Sinova, and T. Jungwirth. Chiral magnons in altermagnetic $RuO_2$. *Phys. Rev. Lett.*, 131(25):256703, 2023.

[28] Z. Liu, M. Ozeki, S. Asai, S. Itoh, and T. Masuda. Chiral split magnon in altermagnetic MnTe. *Phys. Rev. Lett.*, 133(15):156702, 2024.

[29] W. Xing, L. Qiu, X. Wang, Y. Yao, Y. Ma, R. Cai, S. Jia, X. C. Xie, and W. Han. Magnon transport in quasi-two-dimensional van der Waals antiferromagnets. *Phys. Rev. X*, 9(1):011026, 2019.

[30] Q. Cui, B. Zeng, P. Cui, T. Yu, and H. Yang. Efficient spin Seebeck and spin Nernst effects of magnons in altermagnets. *Phys. Rev. B*, 108(18):L180401, 2023.

[31] S. Sarkar and A. Agarwal. Spin-split magnon bands induce pure spin current in insulating altermagnets. *Phys. Rev. B*, 112(19):195420, 2025.

[32] L. V. Pupim and M. S. Scheurer. Noise spectroscopy of insulating and itinerant altermagnets. *arXiv*, 2026. arXiv:2604.23297.

[33] M. Papagno, D. Pacilé, D. Topwal, P. Moras, P. M. Sheverdyaeva, F. D. Natterer, A. Lehnert, S. Rusponi, Q. Dubout, F. Calleja, E. Frantzeskakis, S. Pons, J. Fujii, I. Vobornik, M. Grioni, C. Carbone, and H. Brune. Two distinct phases of bilayer graphene films on Ru(0001). *ACS Nano*, 6(10):9299–9304, 2012.

[34] P. Ouyang, J. Yu, Q. Li, G. Jia, Y. Wang, K. Xiao, H. Zhang, Z. Hu, P. A. Pantaleón, Z. Zhan, S. Zhou, F. Guinea, Q.-K. Xue, and W. Li. Structural and electronic signatures of strain-tunable marginally twisted bilayer graphene. *Natl. Sci. Rev.*, 13(4):nwaf568, 2026.
